# Prediction of Deflection of Reinforced Concrete Beams Strengthened with Fiber Reinforced Polymer

**DOI:** 10.3390/ma12091367

**Published:** 2019-04-26

**Authors:** Mykolas Daugevičius, Juozas Valivonis, Tomas Skuturna

**Affiliations:** Department of Reinforced Concrete Structures and Geotechnics, Vilnius Gediminas Technical University, LT-10223 Vilnius, Lithuania; juozas.valivonis@vgtu.lt (J.V.); tomas.skuturna@vgtu.lt (T.S.)

**Keywords:** strengthening, FRP, deflection, yielding, effective moment of inertia

## Abstract

The article analyses the calculation of the deflection of reinforced concrete beams strengthened with fiber reinforced polymer. This paper specifically focuses on estimating deflection when the yielding of reinforcement is reached. The article proposes a simple method for calculating deflection that was compared with the experimentally predicted deflection. The carried out comparison has showed that the proposed method is suitable not only for the strengthened beams but also for the reinforced concrete beams with a varying reinforcement ratio. The suggested calculation method is based on the effective moment of inertia, such as the one introduced in the ACI Committee 318 Building Code Requirement for Structural Concrete (ACI318). The development of deflection was divided into three stages, and equations for the effective moment of inertia were proposed considering separate stages. In addition, the put forward equations were modified attaching additional relative coefficients evaluating a change in the depth of the neutral axis.

## 1. Introduction

One of the greatest advantages that can provide strengthening with carbon fiber reinforced polymer (CFRP) is an increase in the flexibility of the beam. Failure in the reinforced concrete beam is related to steel yielding, concrete crashing, or shear failure. Short-term and long-term experiments have showed that strengthening RC beams with CFRP can delay steel yielding [1,2,3,4,5,6]. Evenly, if steel yielding is reached or steel is rusted, the strengthened beams can serve until the rupture, delamination of the CFRP layer, steel fatigue fracture, or concrete crashing are achieved [7,8,9,10,11]. Due to high strength and high elasticity, the tensioned layer of CFRP can intercept tensile forces (stresses) when the yielding of reinforcement is reached. That is why the deflection of the beam can develop, thus reaching the yielding of reinforcement at a later stage. However, there is a danger for premature debonding of CFRP layer. In order to prevent this, proper additional anchoring can delay this phenomenon [12]. As well near surface mounted CFRP due to a larger perimeter-to-sectional-area-ratio can ensure better bond performance [13].

Various researches demonstrate that deflection development and reached yielding depend on the reinforcement (steel) ratio [14,15]. This may be related to the exploitation of the compressed concrete. If the reinforcement ratio is low, the exploitation of the compressed concrete is also greatly reduced until the yielding of reinforcement is reached. Therefore, the deflection (when the yielding of reinforcement is reached) of the strengthened beams with a low reinforcement ratio is the biggest. This is due to the unexploited deformability of the compressed concrete.

The existing methods for calculating deflection can perform estimation until the yielding of reinforcement is reached. The most common and simplest methods are based on design guidelines ACI318 [16] and the Eurocode 2 [17]. In addition, the multi-layer method can be used for calculating the deflection of the strengthened beams; however, this method is not that convenient for engineers, and therefore will not be discussed in this article. The calculation method based on ACI318 [16] evaluates the effective moment of inertia, and the method based on Eurocode 2 [17], usually evaluates the average curvature of the bending element. Both methods evaluate the moment of the inertia of the full cross-section and the moment of the inertia of the cross-section where the crack is opened. However, these methods evaluate stress strain state in the cross-section before yield stresses in reinforcement are reached. There are several methods [18,19,20,21] that can evaluate stress-strain state in the cross-section after yield stresses are reached but these methods are difficult to be applied by the designer. Several contributions based on the moment-curvature modeling are available [22,23]. The accuracy of the proposed model [22,23] is impressive, however certain parameters like moment of inertia, depth of the neutral axis remains unknown.

The load carrying capacity of the strengthened beams can significantly increase such that the increased service load can locate in the range of the load-deflection curve where steel yielding is reached. The main objective of this article is to calculate the deflection of the strengthened beam when steel yielding is reached and when only the layer of CFRP intercepts tensile forces.

## 2. Analyzed Beams

RC strengthened beams with various reinforcement ratios were chosen to perform the calculation of deflection. The data about beams were collected from various research. The references and titles of the analyzed beams with a short description are presented in Table 1. The chosen beams are suitable for deflection analysis, because deflection develops when the yielding of reinforcement is reached. As mentioned above, a lower reinforcement ratio allows a higher increment in deflection when the yielding of reinforcement is reached.

The mechanical parameters of the material such as the modulus of elasticity and tensile strength are required in order to calculate the deflection of the beam. This and other mechanical parameters are presented in Table 2.

## 3. Calculation of Deflection

**Figure 1:**
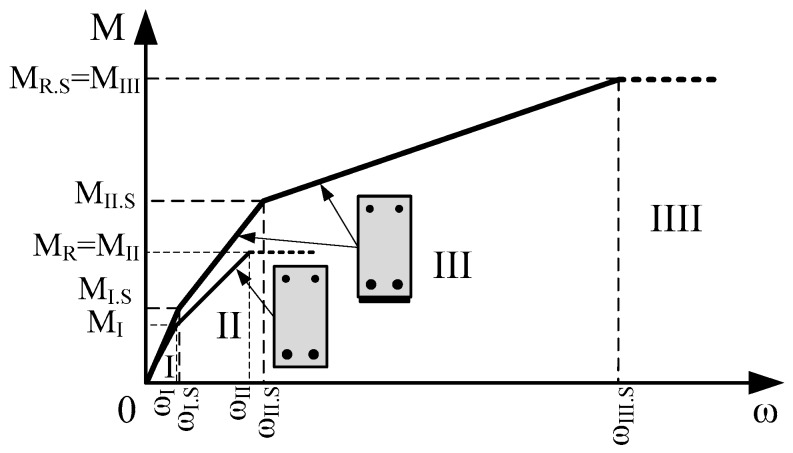
The development of the deflection of the strengthened and unstrengthened beam.

The development of the deflection of the strengthened and unstrengthened beams is divided into stages. At the first stage, deflection develops until vertical cracks open in the tensioned part of the cross-section. At the second stage, deflection develops when the vertical crack is opened until the yielding strength of the tensioned reinforcement is reached. At the third stage, deflection develops when the yielding strength of reinforcement is reached and only a layer of CFRP intercepts tensile force. Therefore, two deflection development stages exist for the unstrengthened beams and three stages for the strengthened ones (Figure 1). Bending moments M_I_ and M_I.S_ are shown in (Figure 1), which is the cracking moment of the unstrengthened and strengthened beam, respectively. Due to the CFRP layer, the contribution cracking moment of the strengthened beam is slightly bigger than that of the unstrengthened beam (M_I.S_ > MI). Bending moments (M_I.S_ and M_I_) correspond to the end of the first stage. The maximal carrying bending moment of the unstrengthened beam (M_R_ = M_II_) is smaller than that of the bending moment of the strengthened beam (M_II.S_) when the yielding of reinforcement is reached. These bending moments correspond to the end of the second stage. The maximum carrying bending moment of the strengthened beam is designated as M_R.S_ = M_III_ and corresponds to the end of the third stage.

**Figure 2:**
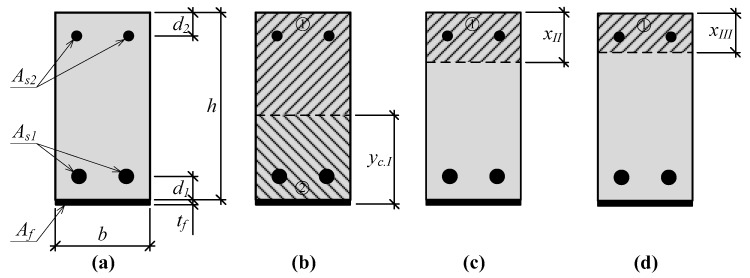
A change in the depth of the neutral axis of the RC strengthened beam: (**a**) Cross-section of the strengthened beam; (**b**) depth of the neutral axis before vertical cracks will open; (**c**) depth of the neutral axis when vertical cracks are opened; (**d**) depth of the neutral axis when steel yielding is reached.

**Figure 3:**
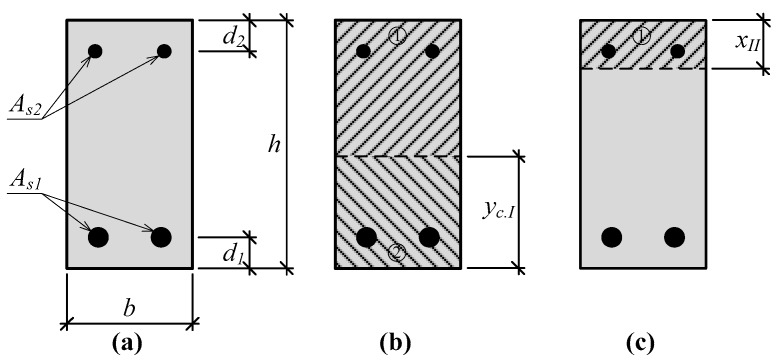
A change in the depth of the neutral axis of the RC beam: (**a**) Cross-section of the beam (**b**) depth of the neutral axis before vertical cracks will open; (**c**) depth of the neutral axis when vertical cracks are opened.

The deflection of the beams at a certain stage is influenced by different flexural stiffness. Generally, bending stiffness *E*·*I* (the product of the modulus of elasticity and the moment of inertia) is influenced by the moment of inertia. The current methods for calculating deflection usually evaluate the modulus of elasticity like for an elastic material. Then, the development of deflection undergoes all stages, cracks in the tensioned part of the cross-section develop, therefore, the moment of the inertia is not constant. Thus, at a certain stage, the depth of the neutral axis and the moment of inertia are different. A change in the depth of the neutral axis of the strengthened and unstrengthened beams is presented in Figure 2 and Figure 3. Thus, there are parts of the cross-section containing and having no cracks. Therefore, the effective moment of inertia should be evaluated. The prediction of the depth of the neutral axis at each stage confirms that the distribution of strains is linear. Stresses in the compressed part of the section are in the elastic range. In addition, a hypothesis about the plane section is valid. The strain of internal and external reinforcement is equal to the surrounded concrete strain (bond slip is not evaluated).

The deflection of the strengthened beam at stage 1 up to the cracking of the tensioned part of the cross-section can be predicted by the equation:

(1) $$\omega_{I.S}\left(M_{I}\right)=\frac{3\cdot l^{2}-4\cdot a^{2}}{24}\cdot \frac{M_{I}}{E_{cm}\cdot I_{I.red}}.$$

where *l*—the span length of the beam, a–distance from the support to the external load position, *M_I_*—acting moment, *E_cm_*—the modulus of elasticity of concrete, *I*_*I*.*red*_—the reduced moment of the inertia of the total cross-section according to the neutral axis of the cross-section.

At stage 1, the evaluated acting moment is 0 < *M_I_* ≤ *M*_*I*.*S*_, and the ultimate bending moment of stage 1 is the cracking moment:

(2) $$M_{crc}=M_{I.S}=f_{ct}\cdot \frac{I_{I.red}}{y_{c.I}}.$$

where *f_ct_*—the tensile strength of concrete, *y*_*c*.*I*_—the centre of the gravity of the cross-section at stage 1. The center of gravity can be predicted by the following equations:

(3) $$A_{red}=b\cdot h+\alpha_{f}\cdot A_{f}+\left(\alpha_{s1}-1\right)\cdot A_{s1}+\left(\alpha_{s2}-1\right)\cdot A_{s2},$$

(4) $$S_{red}=b\cdot h\cdot \left(\frac{h}{2}+t_{f}\right)+\alpha_{f}\cdot A_{f}\cdot \frac{t_{f}}{2}+\left(\alpha_{s1}-1\right)\cdot A_{s1}\cdot \left(d_{1}+t_{f}\right)+\left(\alpha_{s2}-1\right)\cdot A_{s2}\cdot \left(h+t_{f}-d_{2}\right),$$

(5) $$\alpha_{f}=\frac{E_{f}}{E_{c}},$$

(6) $$\alpha_{s1}=\frac{E_{s1}}{E_{c}},$$

(7) $$\alpha_{s2}=\frac{E_{s2}}{E_{c}},$$

(8) $$y_{c.I}=\frac{S_{red}}{A_{red}}.$$

where *A_red_*—the reduced cross-section of the strengthened beam, *A_f_*—the cross section of carbon fibers, *A_s_*_1_, *A_s_*_2_—the cross-section of steel bars, *S_red_*—the static moment of the reduced cross-section of the strengthened beam, *α_f_*, *α_s_*_1_, *α_s_*_2_—coefficients of reduction, *E_f_*—the modulus of elasticity of fibers, *E_s_*_1_, *E_s_*_2_—the modulus of elasticity of the steel bars.

The reduced moment of the inertia of the cross-section can be predicted by the following equation:

(9) $$\begin{array}{l} I_{I.red}=\frac{b\cdot h^{3}}{12}+b\cdot h\cdot {\left(\frac{h}{2}+t_{f}-y_{c.I}\right)}^{2}+\alpha_{f}\cdot A_{f}\cdot {\left(y_{c.I}-\frac{t_{f}}{2}\right)}^{2}\\ +\left(\alpha_{s1}-1\right)\cdot A_{s1}\cdot {\left(y_{c.I}-t_{f}-d_{1}\right)}^{2}+\left(\alpha_{s2}-1\right)\cdot A_{s2}\cdot {\left(h+t_{f}-y_{c.I}-d_{2}\right)}^{2}. \end{array}$$

The deflection of the strengthened beam at stage 2, when the tensioned part of the cross-section is cracked and the yielding of the tensioned reinforcement is not reached, can be predicted by the equation:

(10) $$\omega_{II}\left(M_{II}\right)=\frac{3\cdot l^{2}-4\cdot a^{2}}{24}\cdot \frac{M_{II}}{E_{c}\cdot I_{II}\left(M_{II}\right)}.$$

The acting bending moment at stage 2 is *M_II_* and the moment *M*_*I*.*S*_ < *M_II_* ≤ *M*_*II*.*S*_. The moment when the yielding of reinforcement is reached is *M*_*II*.*S*_. The effective moment of inertia is evaluated using the Branson [45] equation for parameter *I_II_*:

(11) $$I_{II}\left(M_{II}\right)=I_{I.red}\cdot {\left(\frac{M_{I.u}}{M_{II}}\right)}^{3}+I_{II.red}-I_{II.red}\cdot {\left(\frac{M_{I.u}}{M_{II}}\right)}^{3}.$$

If change of the neutral axis is evaluated, then Equation (11) is modified like:

(12) $$I_{II}\left(M_{II}\right)=I_{I.red}\cdot {\left(\frac{M_{I.u}}{M_{II}}\right)}^{3}+I_{II.red}\cdot \gamma_{1.c}\cdot \gamma_{1.t}-I_{II.red}\cdot {\left(\frac{M_{I.u}}{M_{II}}\right)}^{3}\cdot \gamma_{1.c}\cdot \gamma_{1.t}.$$

where *I*_*II*.*red*_—the reduced moment of the inertia of the cross section where the vertical crack is opened. This moment of inertia can be predicted by the equation:

(13) $$\begin{array}{l} I_{II.red}=\frac{b\cdot x_{II}^{3}}{12}+b\cdot x_{II}\cdot {\left(\frac{x_{II}}{2}\right)}^{2}+\alpha_{f}\cdot A_{f}\cdot {\left(h+t_{f}-x_{II}-\frac{t_{f}}{2}\right)}^{2}\\ +\alpha_{s1}\cdot A_{s1}\cdot {\left(h-x_{II}-d_{1}\right)}^{2}+\left(\alpha_{s2}-1\right)\cdot A_{s2}\cdot {\left(x_{II}-d_{2}\right)}^{2}. \end{array}$$

Coefficients *γ*_1.*c*_ and *γ*_1.*t*_ evaluate a change in the neutral axis and can be predicted by equations:

(14) $$\gamma_{1.c}=\frac{x_{II}}{x_{I}},$$

(15) $$\gamma_{1.t}=\frac{h+t_{f}-x_{II}}{h+t_{f}-x_{I}}.$$

The depth of the neutral axis at stage 1 is predicted by the equation:

(16) $$x_{I}=h+t_{f}-y_{c.I}.$$

**Figure 4:**
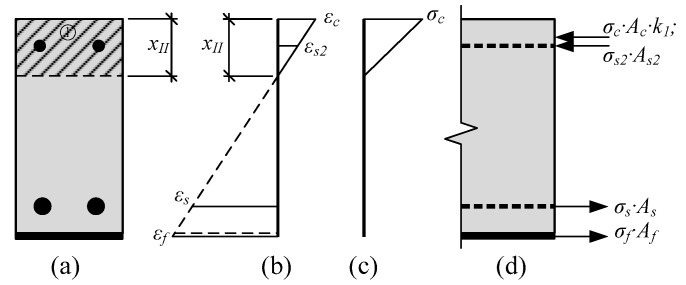
Stress-strain state in the strengthened RC beam until the yielding of reinforcement is reached: (**a**) Depth of the neutral axis; (**b**) distribution of strains; (**c**) distribution of stresses; (**d**) internal forces.

The prediction of the depth of the neutral axis in the section having an opened crack is based on the previously mentioned assumptions. The hypothesis of plain sections is valid. The distribution of strains through the height of the section is linear (Figure 4b). Then, by the similarity of triangles, strains at each layer, in proportion with the strain of the compressed concrete layer, can be expressed, and the depth of the neutral axis should be expressed from the square equation. The depth of the neutral axis at stage 2 can be predicted by the equation:

(17) $$x_{II}=\frac{-B+\sqrt{B^{2}+4\cdot A\cdot C}}{2\cdot A}.$$

where coefficients *A*, *B*, and *C*:

(18) A=b⋅0.5,

(19) $$B=\alpha_{f}\cdot A_{f}+\alpha_{s1}\cdot A_{s1}+\left(\alpha_{s2}-1\right)\cdot A_{s2},$$

(20) $$C=\alpha_{f}\cdot A_{f}\cdot \left(h+\frac{t_{f}}{2}\right)+\alpha_{s1}\cdot A_{s1}\cdot d+\left(\alpha_{s2}-1\right)\cdot A_{s2}\cdot d_{2}.$$

The deflection of the strengthened beam at stage 3, when the yielding strength of tensioned reinforcement is reached, can be predicted by the equation:

(21) $$\omega_{III}\left(M_{III}\right)=\frac{3\cdot l^{2}-4\cdot a^{2}}{24}\cdot \frac{M_{III}}{E_{c}\cdot I_{III}\left(M_{III}\right)}.$$

The acting bending moment at stage 3 is *M_III_* and the moment *M*_*II*.*u*_ < *M_III_* ≤ *M*_*III*.*u*_. The ultimate bending moment at stage 3 is *M*_*III*.*u*_. The new effective moment of inertia is evaluated in the equation for parameter *I_III_*:

(22) $$\begin{array}{l} I_{III}\left(M_{III}\right)=I_{I.red}\cdot {\left(\frac{M_{I.u}}{M_{III}}\right)}^{3}+I_{II.red}\cdot {\left(\frac{M_{II.u}}{M_{III}}\right)}^{3}-I_{II.red}\cdot {\left(\frac{M_{I.u}}{M_{III}}\right)}^{3}\\ +I_{III.red}\cdot {\left(\frac{M_{III}}{M_{III}}\right)}^{3}-I_{III.red}\cdot {\left(\frac{M_{II.u}}{M_{III}}\right)}^{3}. \end{array}$$

If change of the neutral axis is evaluated, then Equation (22) is modified like:

(23) $$\begin{array}{l} I_{III}\left(M_{III}\right)=I_{I.red}\cdot {\left(\frac{M_{I.u}}{M_{III}}\right)}^{3}+I_{II.red}\cdot {\left(\frac{M_{II.u}}{M_{III}}\right)}^{3}\cdot \gamma_{1.c}\cdot \gamma_{1.t}-I_{II.red}\cdot {\left(\frac{M_{I.u}}{M_{III}}\right)}^{3}\cdot \gamma_{1.c}\cdot \gamma_{1.t}\\ +I_{III.red}\cdot {\left(\frac{M_{III}}{M_{III}}\right)}^{3}\cdot \gamma_{2.c}\cdot \gamma_{2.t}-I_{III.red}\cdot {\left(\frac{M_{II.u}}{M_{III}}\right)}^{3}\cdot \gamma_{2.c}\cdot \gamma_{2.t}. \end{array}$$

where *I*_*III*.*red*_—the reduced moment of the inertia of the cross section where the vertical crack is opened. This moment of inertia can be predicted by the equation:

(24) $$\begin{array}{l} I_{III.red}=\frac{b\cdot x_{III}^{3}}{12}+b\cdot x_{III}\cdot {\left(\frac{x_{III}}{2}\right)}^{2}+\alpha_{f}\cdot A_{f}\cdot {\left(h+t_{f}-x_{III}-\frac{t_{f}}{2}\right)}^{2}\\ +\left(\alpha_{s2}-1\right)\cdot A_{s2}\cdot {\left(x_{III}-d_{2}\right)}^{2}. \end{array}$$

Coefficients *γ*_2.*c*_ and *γ*_2.*t*_:

(25) $$\gamma_{2.c}=\frac{x_{III}}{x_{II}};$$

(26) $$\gamma_{2.t}=\frac{h+t_{f}-x_{III}}{h+t_{f}-x_{II}}$$

**Figure 5:**
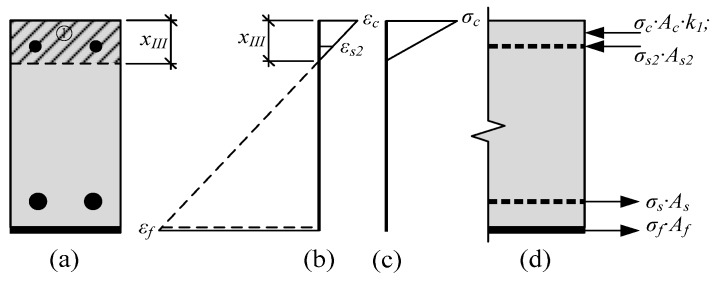
Stress-strain state in the strengthened RC beam when the yielding of reinforcement is reached: (**a**) Depth of the neutral axis; (**b**) distribution of strains; (**c**) distribution of stresses; (**d**) internal forces.

The depth of the neutral axis at stage 3 is also predicted from the similarity of triangles (Figure 5b).

The depth of the neutral axis at stage 3 is predicted by the equation:

(27) $$x_{III}=\frac{-B+\sqrt{B^{2}+4\cdot A\cdot C}}{2\cdot A}.$$

Were coefficients *A*, *B*, and *C*:

(28) A=b⋅0.5;

(29) $$B=\alpha_{f}\cdot A_{f}+\left(\alpha_{s2}-1\right)\cdot A_{s2};$$

(30) $$C=\alpha_{f}\cdot A_{f}\cdot \left(h+\frac{t_{f}}{2}\right)+\left(\alpha_{s2}-1\right)\cdot A_{s2}\cdot d_{2}.$$

The deflection of the unstrengthened beams can be predicted by the same Equations (1) and (10). However, the parameters of the FRP layer in other equations should be ignored. If the beams are strengthened with the prestressed FRP, in this case it is necessary to calculate the additional curvature and the deflection from prestress force. The total deflection is obtained by summing up all the deflections.

## 4. Results

**Figure 6:**
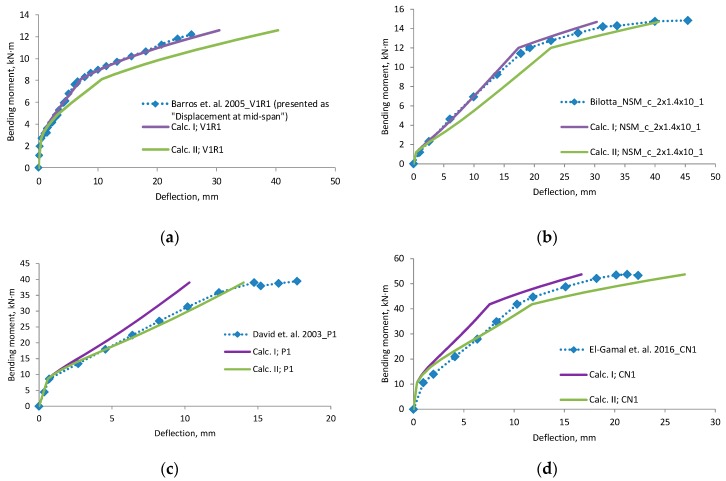
Bending moment–deflection curves, (**a**) beam V1R1; (**b**) beam NSM_c_2 × 1.4 × 10_1; (**c**) beam P1; (**d**) beam CN1.

**Figure 7:**
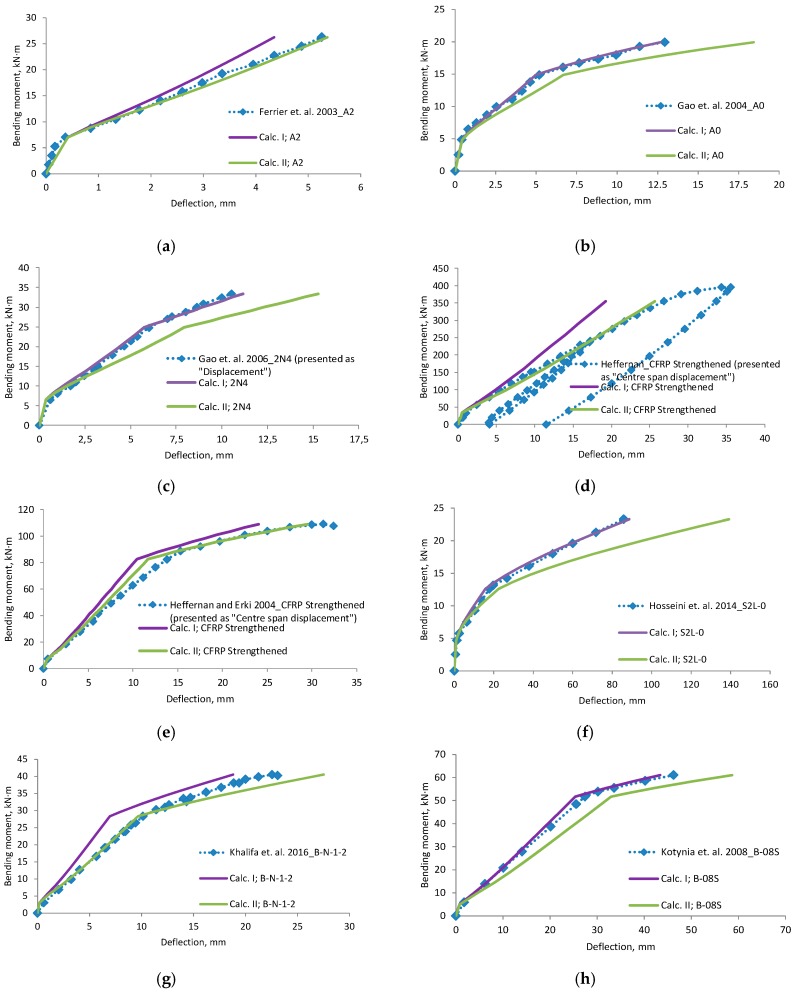
Bending moment–deflection curves, (**a**) beam A2; (**b**) beam A0; (**c**) beam 2N4; (**d**) beam CFRP Strengthened; (**e**) beam CFRP strengthened; (**f**) beam S2L-0; (**g**) beam B-N-1-2; (**h**) beam B-08S.

**Figure 8:**
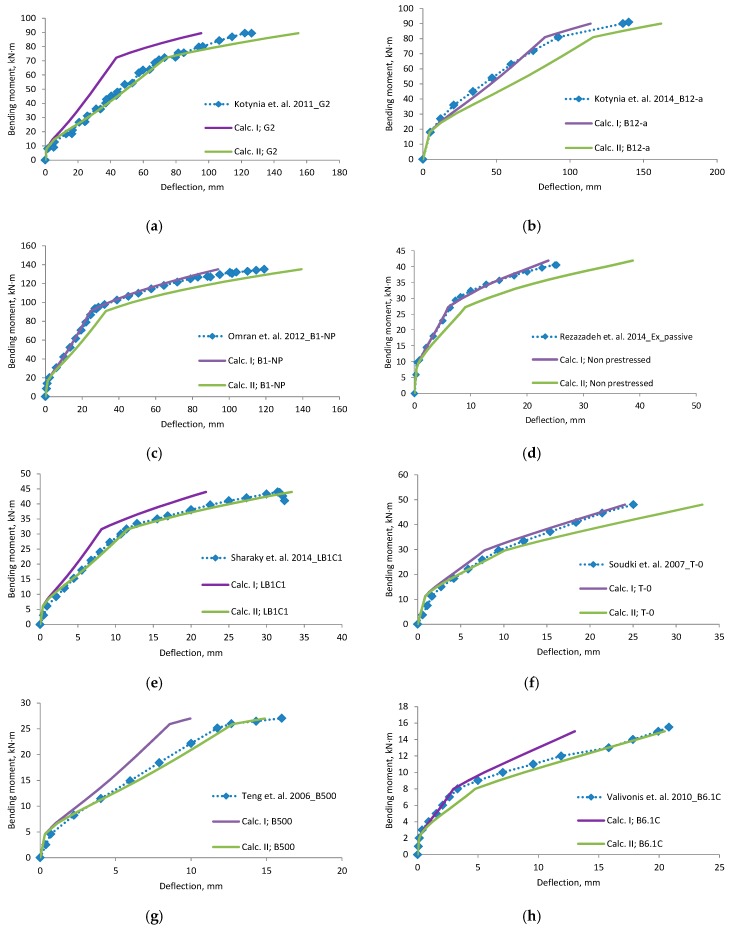
Bending moment–deflection curves, (**a**) beam G2; (**b**) beam B12-a; (**c**) beam B1-NP; (**d**) beam Non prestressed, (**e**) beam LB1C1; (**f**) beam T-0; (**g**) B500; (**h**) B6.1C.

**Figure 9:**
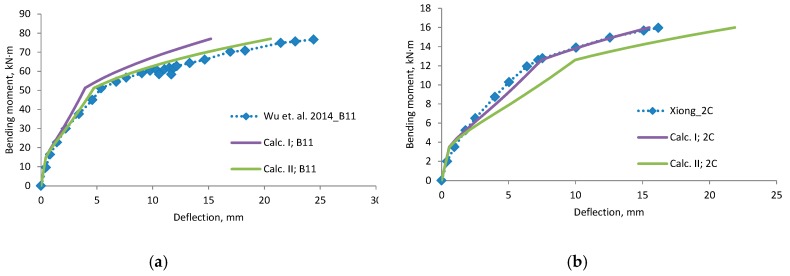
Bending moment–deflection curves, (**a**) beam B11; (**b**) beam 2C.

A comparison of deflections (Figure 6, Figure 7, Figure 8 and Figure 9) shows that the equation method is suitable for RC beams with various reinforcement ratios. Calculated deflections of all mentioned beams are presented in the Appendix A. In these figures, designation “Calc. I” is related to Equations (11) and (22). Designation “Calc. II” related with Equations (12) and (23). It is clear that the theoretical equation method gives brake points such as the cracking moment and steel yielding moment on the load deflection curve. The difference between the calculated and experimental deflection increases when the load level increases. This may happen because the theoretical method evaluates the elastic work of concrete and the constant depth of the neutral axis. Thus, the deflection curve curvature depends just from ratio of the bending moments. In order to increase the accuracy of the theoretical method, nonlinear stress-strain distribution across the height of the cross-section should be evaluated. The proposed method evaluates linear stress-strain distribution. The evaluation of nonlinear stress-strain distribution can be complex for designers, and thus triangular distribution is easier to assess. Furthermore, a comparison of the position of the center of the parabolic and triangular form gives little difference. The difference in results is also influenced by the accuracy of the experiment. In certain experiments, deflection at the cracking moment to big. The main drawback of the suggested method is the prediction of the bending moment when steel yielding is reached. It is difficult to predict the moment when the FRP layer is incorporated, because strains are not known in the compressed concrete and tensioned CFRP layer. In such a case, the problem must be solved by the iteration approach until the balance of internal forces is reached. This is also a complex task for designers. For this research values of cracking, yielding and ultimate moment were predicted from the deflection evolution plots.

Experiments in which the deflection was measured from the frame mounted on a beam gives a more precise result. Calculated deflection (Calc. I) using the effective moment of inertia equation without any coefficients is suitable for this measurement system. Equation of the effective moment of inertia must be without coefficients—it is related with the neutral axis. Please note that the second stage does not have a horizontal straight line. The other experimental “deflection“ results, which are more close to the “Calc. II” can be associated with the measured displacement.

## 5. Conclusions

According to the proposed method for calculating the deflection of the strengthened RC beam, it is possible to predict deflection when steel yielding is reached. When the deflection is calculated using the usual expression of an effective moment of inertia (Equations (11) and (22)), in some cases smaller deflections are obtained. This discrepancy may be due to an incorrectly determined experimental deflection, since in some experiments it is not clear whether the deflection is determined by compensating the lift of the neutral axis at the supports. In most cases, the most accurate calculation using the normal expression of an effective inertia moment (Equations (11) and (22)). Estimating the change in the neutral axis (Equations (12) and (23)) results in bigger deflections but are more precise when the deflections are lower with normal expression (Equations (11) and (22)). Another important criterion related to the accuracy of deflections is the coefficient of estimating the nature of the external load, since after the strengthening the evolution of cracks changes, the curvature development change too. In order to verify the accuracy of the experimental and computational results, further finite element analysis is required.

## Figures and Tables

**Table 1:** Characteristics of investigated experimental beams.

Author	Beam Name	l, m	Load Positions, m	b, m	h, m	A_s1_	A_s2_	d_1_, m	d_2_, m	A_f_
Barros et al., 2005 [24]	V1	1.5	0.5 + 0.5 + 0.5	0.1	0.178	2Ø6	2Ø8	0.024	0.025	–
	V1R1				0.17	2Ø6				1 × 1.45 × 9.59
	V2				0.173	3Ø6				–
	V2R2				0.177	3Ø6				2 × 1.45 × 9.59
	V3				0.175	2Ø6				–
	V3R2				0.175	2Ø6 + Ø8				2 × 1.45 × 9.59
	V4				0.175	3Ø8		0.025		–
	V4R3				0.18	3Ø8				3 × 1.45 × 9.59
Bilotta et al., 2015 [25]	Ref_c_no_1	2.1	0.925 + 0.25 + 0.925	0.12	0.16	2Ø10	2Ø10	0.05	0.035	–
	Ref_d_no_1		Distributed load							–
	EBR_c_1.4 × 40_1		0.925 + 0.25 + 0.925							56 mm^2^
	EBR_c_1.4 × 40_2									56 mm^2^
	EBR_d_1.4 × 40_1		Distributed load							56 mm^2^
	EBR_d_1.4 × 40_2									56 mm^2^
	NSM_c_2_1.4 × 10_1		0.925 + 0.25 + 0.925							28 mm^2^
	NSM_d_2_1.4 × 10_1		Distributed load							28 mm^2^
	NSM_c_3_1.4 × 10_1		0.925 + 0.25 + 0.925							42 mm^2^
	NSM_d_3_1.4 × 10_1		Distributed load							42 mm^2^
David et al., 2003 [26]	P1	2.8	0.9 + 1.0 + 0.9	0.15	0.3	2Ø14	2Ø8	0.027	0.024	–
	P2									1.2 (cm^2^)
	P5									2.4 (cm^2^)
EL-Gamal et al., 2016 [27]	REF	2.36	0.93 + 0.5 + 0.93	0.2	0.3	2Ø12	2Ø8	0.04	0.032	–
	CN1									71.26 (mm^2^)
	CN2									2 × 71.26 (mm^2^)
	GN1									71.3 (mm^2^)
	GN2									2 × 71.3 (mm^2^)
	CHYB									71.26 + 25.8 (mm^2^)
	GHYB									71.3 + 25.8 (mm^2^)
	REF-II					4Ø12				–
	CN1-II									71.26 (mm^2^)
	CN2-II									2 × 71.26 (mm^2^)
Ferrier et al., 2003 [28]	A1	2.0	0.7 + 0.6 + 0.7	0.15	0.25	2Ø14	2Ø8	0.025	0.025	–
	A2									120 (mm^2^)
Gao et al., 2004 [29]	CON1	1.5	0.5	0.15	0.2	2Ø10	2Ø8	0.038	0.027	–
	A0									0.22 × 75
	A10									0.22 × 75
	A20									0.22 × 75
	B0									0.44 × 75
	B10									0.44 × 75
	B20									0.44 × 75
Gao et al., 2006 [30]	2O	1.5	0.5	0.15	0.2	2Ø10	2Ø8	0.038	0.027	–
	2N6									6 × 0.11 × 150
	2T625-1									
	2T650-1									
	2T675-1									
	2N4									4 × 0.11 × 150
	2T450-1									
	2T4100-1									
Heffernan 1997 [31]	Conventional	4.8	1.6 + 1.6 + 1.6	0.3	0.5739	2Ø25 + Ø20	2Ø10	0.074	0.067	–
	CFRP strengthened									65.5 (mm^2^)
Heffernan and Erki 2004 [32]	Conventional	2.85	1.1 + 0.65 + 1.1	0.15	0.3	2Ø20 + Ø10	2Ø10	0.041	0.037	–
	CFRP strengthened									89.4 (mm^2^)
Hosseini et al., 2014 [33]	SREF	2.4	0.9 + 0.6 + 0.9	0.6	0.12	4Ø8	3Ø6	0.024	0.023	–
	S2L-0									2 × 1.4 × 20
	S2L-20									
	S2L-40									
Khalifa et al., 2016 [34]	B-C	2.2	0.95 + 0.3 + 0.95	0.15	0.26	2Ø12	2Ø12	0.041	0.031	–
	B-S-2									60 (mm^2^)
	B-S-4									120 (mm^2^)
	B-N-1-2									60 (mm^2^)
	B-N-2-2									60 (mm^2^)
	B-N-2-4									120 (mm^2^)
Kotynia et al., 2008 [35]	B-08S	4.2	1.4 + 1.4 + 1.4	0.15	0.3	3Ø12	2Ø10	0.03 *	0.03 **	60 (mm^2^)
	B-083m									58.5 (mm^2^)
Kotynia et al., 2011 [36]	G1	6.0	1.2 + 1.2 + 1.2 + 1.2 + 1.2	1.0	0.22	7Ø12	7Ø8	0.03143 *	0.024 **	–
	G2									120 (mm^2^)
	G3									120 (mm^2^)
	G4									120 (mm^2^)
Kotynia et al., 2014 [37]	B12-a	6.0	1.2 + 1.2 + 1.2 + 1.2 + 1.2	0.5	0.22	4Ø12	4Ø8	0.031	0.029	1.2 × 100
	B12-asp									1.2 × 100
	B16-asp									1.2 × 100
Omran et al., 2012 [38]	B0	5.0	2 + 1 + 2	0.2	0.4	3Ø15	2Ø10	0.057	0.036	–
	B1-NP									2 × 2 × 16
	B1-P1									
	B1-P2									
	B1-P3									
Rezazadeh et al., 2014 [39]	Control	2.2	0.9 + 0.4 + 0.9	0.15	0.3	2Ø10	2Ø10	0.035	0.025	–
	Non prestressed									1.4 × 20
	20% prestressed									
	30% prestressed									
	40% prestressed									
Sharaky et al., 2014 [40]	CB	2.4	0.8 + 0.8 + 0.8	0.16	0.28	2Ø12	2Ø8	0.036	0.034	–
	LB1C1									1Ø8
	LB1G1									1Ø8
	LB2C1									2Ø8
	LB2G1									2Ø8
	LA2C1									2Ø8
	LA2G1									2Ø8
	LB1G2									1Ø12
Soudki et al., 2007 [41]	C-0	2.25	0.75	0.15	0.25	2Ø10	2Ø6	0.025	0.023	–
	T-0									4 × 0.11
	S-0									50 × 1.2
Teng et al., 2006 [42]	B0	3.0	1.2 + 0.6 + 1.2	0.15	0.3	2Ø12	2Ø8	0.036	0.034	–
	B500									2 × 16
	B1200									
	B1800									
	B2900									
Valivonis et al., 2010 [14]	B6.1C	1.2	0.4 + 0.4 + 0.4	100	200	2Ø6	2Ø6	0.025	0.025	0.167 (cm^2^)
	B6.2C									
	B6.5									–
	B8.1C					2Ø8				0.167 (cm^2^)
	B8.2C									
	B8.3									–
	B12.1C				203	2Ø12	2Ø8			0.167 (cm^2^)
	B12.2C				200					
	B12.5			104	198					–
	B12.6			105	201					
Wu et al., 2014 [43]	Control	1.8	0.6 + 0.6 + 0.6	0.15	0.3	3Ø14	2Ø6	0.037	0.033	–
	B11									Ø7.9
	B21									2Ø7.9
	B22									
	BP11									Ø7.9
	BP12									
	BP13									
	BP14									
Xiong et al., 2007 [44]	Pa	2.1	0.7	0.125	0.2	2 × 10	2×8	0.03	0.024	–
	2C									0.22 × 100
	Pb					2 × 12		0.031		–

* a_s_ = h-A_s1_/ń_s1_·b; ** evaluated individually; l—span length; b—total width of the beam; h—height of the beam; A_s1_—cross-section of the tensioned steel bars; A_s2_—cross-section of the compressed steel bars; d_1_—position of the tensioned steel bars; d_2_—position of the compressed steel bars; A_f_—cross-section of the tensioned fibers or FRP; ńs1—reinforcement ratio by A_s1_.

**Table 2:** Mechanical characteristics of investigated experimental beams materials.

Author	Beam Name	f_c_, MPa	f_ct_, MPa	E_c_, GPa	f_y1_, MPa	f_y2_, MPa	E_s1_, GPa	E_s2_, GPa	f_f,fe_, MPa	E_f,fe_, GPa
Barros et al., 2005 [24]	V1	46.1	3.37	33.35	730	554.32	200	200	–	–
	V1R1								2740	158.8
	V2	46.1	3.58	36.5	730				–	–
	V2R2								2740	158.8
	V3	46.1	3.21	34.89	730				–	–
	V3R2				730; 554.32				2740	158.8
	V4	46.1	3.43	35.86	554.32				–	–
	V4R3								2740	158.8
Bilotta et al., 2015 [25]	Ref_c_no_1	17.4	1.34	25.98	540	540	200	200	–	–
	Ref_d_no_1								–	–
	EBR_c_1.4 × 40_1								2052	171
	EBR_c_1.4 × 40_2									
	EBR_d_1.4 × 40_1									
	EBR_d_1.4 × 40_2									
	NSM_c_2_1.4 × 10_1									
	NSM_d_2_1.4 × 10_1									
	NSM_c_3_1.4 × 10_1									
	NSM_d_3_1.4 × 10_1									
David et al., 2003 [26]	P1	38.7	2.94 ^1^	33.02 ^2^	500	500	205 ^3^	205 ^3^	–	–
	P2	39.2	2.97 ^1^	33.14 ^2^					2400	150
	P5	40.1	3.03 ^1^	33.37 ^2^						
EL-Gamal et al., 2016 [27]	REF	49.62	2.99	35.57 ^2^	480	455	205 ^3^	205 ^3^	–	–
	CN1								1588	119.4
	CN2									
	GN1								1185	52.34
	GN2									
	CHYB								2096 *	147.47 *
	GHYB								1800 *	98.22 *
	REF-II								–	–
	CN1-II								1588	119.4
	CN2-II									
Ferrier et al., 2003 [28]	A1	39	2.96 ^1^	31	550	550 ^3^	210	210 ^3^	–	–
	A2								650	80
Gao et al., 2004 [29]	CON1	35.7	2.75 ^1^	25	531	400	200	200	–	–
	A0								4200	235
	A10									
	A20									
	B0									
	B10									
	B20									
Gao et al., 2006 [30]	2O	62.1	4.29 ^1^	37.1	460	460	200	205	–	–
	2N6								4200	235
	2T625-1									
	2T650-1									
	2T675-1									
	2N4									
	2T450-1									
	2T4100-1									
Heffernan 1997 [31]	Conventional	32.9	2.56 ^1^	31.45 ^2^	-	-	200	200	–	–
	CFRP strengthened									325
Heffernan and Erki 2004 [32]	Conventional	37	2.83 ^1^	32.57 ^2^	511 & 411	411	210	210	–	–
	CFRP strengthened									233
Hosseini et al., 2014 [33]	SREF	46.7	3.43 ^1^	29.7	486	464	200	200	–	–
	S2L-0								2483.9	153.2
	S2L-20									
	S2L-40									
Khalifa et al., 2016 [34]	B-C	35	2.7 ^1^	28	400	400	200	200	2800	165
	B-S-2									
	B-S-4									
	B-N-1-2									
	B-N-2-2									
	B-N-2-4									
Kotynia et al., 2008 [35]	B-08S	32.3	2.52 ^1^	31.27 ^2^	490	524	195	209	2915	172
	B-083m	34.4	2.66 ^1^	31.87 ^2^	436	524	220	209	3500	230
Kotynia et al., 2011 [36]	G1	45	3.33 ^1^	34.55 ^2^	554	561	200	200	–	–
	G2	46.2	3.4 ^1^	34.82 ^2^					2800	165
	G3	45.9	3.39 ^1^	34.75 ^2^						
	G4	45.6	3.37 ^1^	34.68 ^2^					2235	149
Kotynia et al., 2014 [37]	B12-a	45.3	3.35	24.3	539.6	416.2	191.3	186.1	2800	173.3
	B12-asp	32.2	2.51	23.7	511.4	583.2	191.4	200.7		
	B16-asp	49	3.57	25.4	595	555.8	198	196.4		
Omran et al., 2012 [38]	B0	40	3.02 ^1^	27.84	478	500	200	200	–	–
	B1-NP								2610	130.5
	B1-P1									
	B1-P2									
	B1-P3									
Rezazadeh et al., 2014 [39]	Control	32.2	2.51 ^1^	27.4	585	585	208	208	–	–
	Non prestressed								1922	164
	20% prestressed									
	30% prestressed									
	40% prestressed									
Sharaky et al., 2014 [40]	CB	32.4	2.8	31.7	545	545	205	205	–	–
	LB1C1								2350	170
	LB1G1								1350	64
	LB2C1								2350	170
	LB2G1								1350	64
	LA2C1								2350	170
	LA2G1								1350	64
	LB1G2								1350	64
Soudki et al., 2007 [41]	C-0	35	2.7	32.04	460	460	205	205	–	–
	T-0								3480	230
	S-0								2800	165
Teng et al., 2006 [42]	B0	44	3.27 ^1^	34.31 ^2^	–	–	210	210	–	–
	B500								2068	131
	B1200									
	B1800									
	B2900									
Valivonis et al., 2010 [14]	B6.1C	34.4	2.93	32.45	358	358	205	205	4800	231
	B6.2C									
	B6.5								–	–
	B8.1C	29.7	2.63	30.91	557	358	195	205	4800	231
	B8.2C									
	B8.3								–	–
	B12.1C	30.4	2.67	31.14	318	420	204.9	204.1	4800	231
	B12.2C									
	B12.5	28.7	2.56	30.55					–	–
	B12.6								–	–
Wu et al., 2014 [43]	Control	34.4	2.66 ^1^	31.87 ^2^	340	240	200	200	–	–
	B11								2629	170
	B21									
	B22									
	BP11									
	BP12									
	BP13									
	BP14									
Xiong et al., 2007 [44]	Pa	30.71	2.41 ^1^	30.8 ^2^	411	233	200	210	–	–
	2C								3652	252
	Pb				606		210		–	–

^1^ f_ctm_ = 0.3(f_cm_-8)^2/3^ equation from Eurocode 2 [17]; ^2^ E_cm_ = 22(f_cm_/10)^0.3^ equation from Eurocode 2 [17]; ^3^ evaluated individually; f_c_—concrete compressive strength; f_ct_—concrete tensile strength; E_c_—modulus of elasticity of the concrete material; f_y1_—yielding strength of the tensioned steel bars; f_y2_—yielding strength of the compressed steel bars; E_s1_—modulus of elasticity of the tensioned steel bars; E_s2_—modulus of elasticity of the compressed steel bars; f_f,fe_—tensile strength of tensioned fibers or FRP; E_f,fe_—modulus of elasticity tensioned fibers or FRP; *—calculated by the law of the mixture.

**Figure A1:**
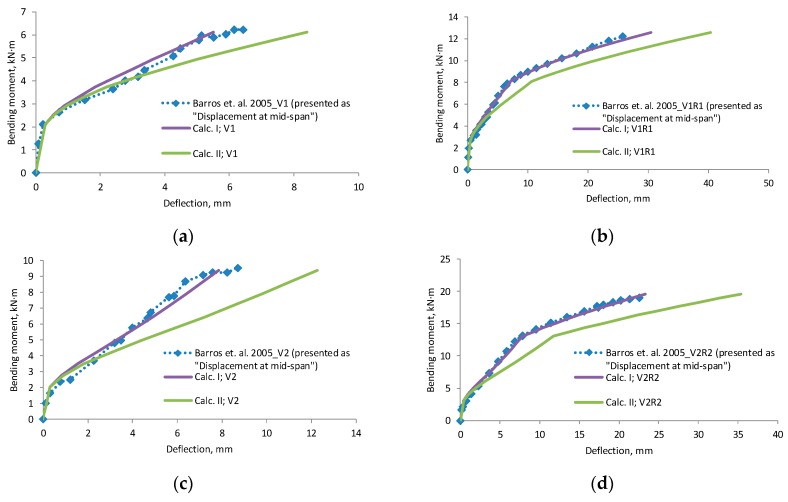

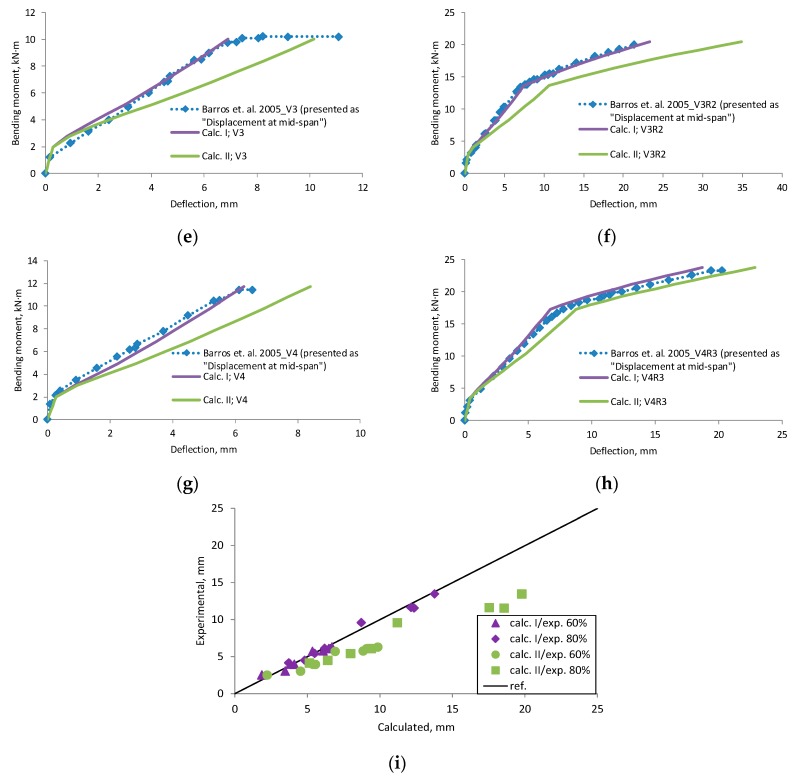
Barros et al., 2005 [24] research beams displacement compared with calculated deflection, (**a**) beam V1; (**b**) beam V1R1; (**c**) beam V2; (**d**) beam V2R2; (**e**) beam V3; (**f**) beam V3R2; (**g**) beam V4; (**h**) beam V4R3; (**i**) scatter of the results at 60% and 80% of the ultimate load.

**Figure A2:**
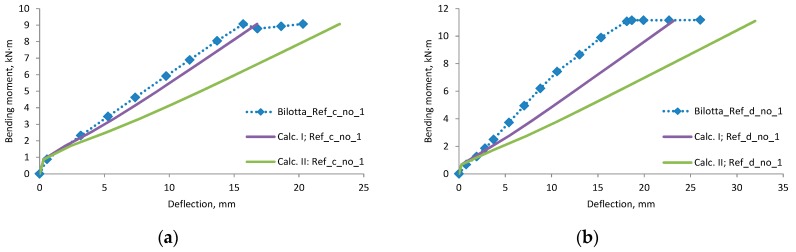

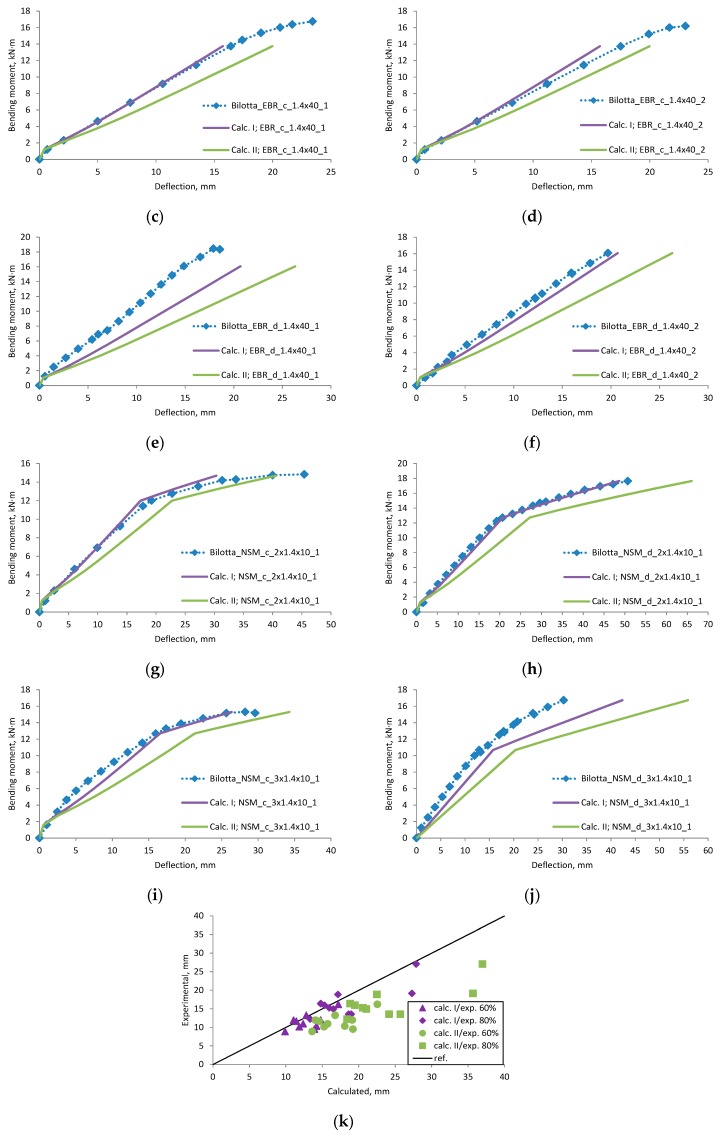
Bilotta et al., 2015 [25] research beams deflection compared with calculated, (**a**) beam Ref_c_no_1; (**b**) beam Ref_d_no_1; (**c**) beam EBR_c_1.4 × 40_1; (**d**) beam EBR_c_1.4 × 40_2; (**e**) beam EBR_d_1.4 × 40_1; (**f**) beam EBR_d_1.4 × 40_2; (**g**) beam NSM_c_2 × 1.4 × 10_1; (**h**) beam NSM_d_2 × 1.4 × 10_1; (**i**) beam NSM_c_3 × 1.4 × 10_1; (**j**) beam NSM_d_3 × 1.4 × 10_1; (**k**) scatter of the results at 60% and 80% of the ultimate load.

**Figure A3:**
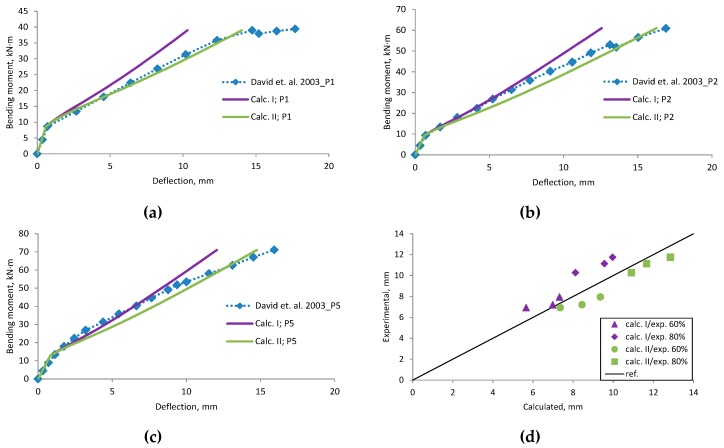
David et al., 2003 [26] research beams deflection compared with calculated, (**a**) beam P1; (**b**) beam P2; (**c**) beam P5; (**d**) scatter of the results at 60% and 80% of the ultimate load.

**Figure A4:**
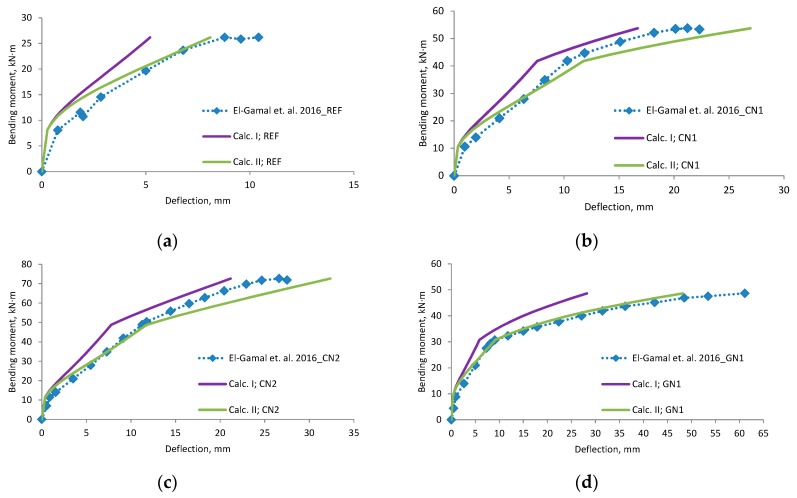

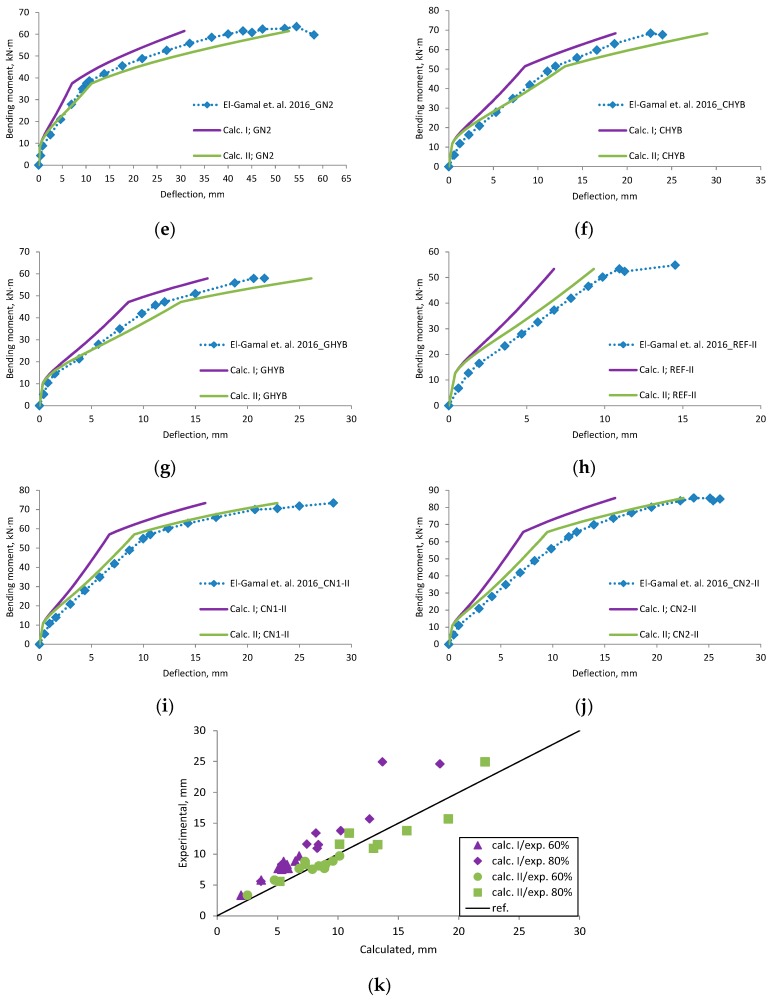
El-Gamal et al., 2016 [27] research beams deflection compared with calculated, (**a**) beam REF; (**b**) beam CN1; (**c**) beam CN2; (**d**) beam GN1; (**e**) beam GN2; (**f**) beam CHYB; (**g**) beam GHYB; (**h**) beam REF-II; (**i**) beam CN1-II; (**j**) beam CN2-II; (**k**) scatter of the results at 60% and 80% of the ultimate load.

**Figure A5:**
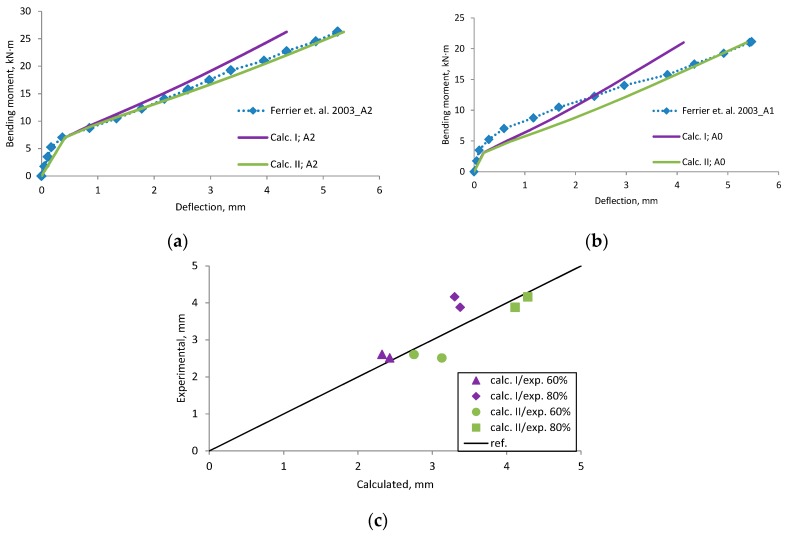
Ferrier et al., 2003 [28] research beams deflection compared with calculated, (**a**) beam A2; (**b**) beam A0; (**c**) scatter of the results at 60% and 80% of the ultimate load.

**Figure A6:**
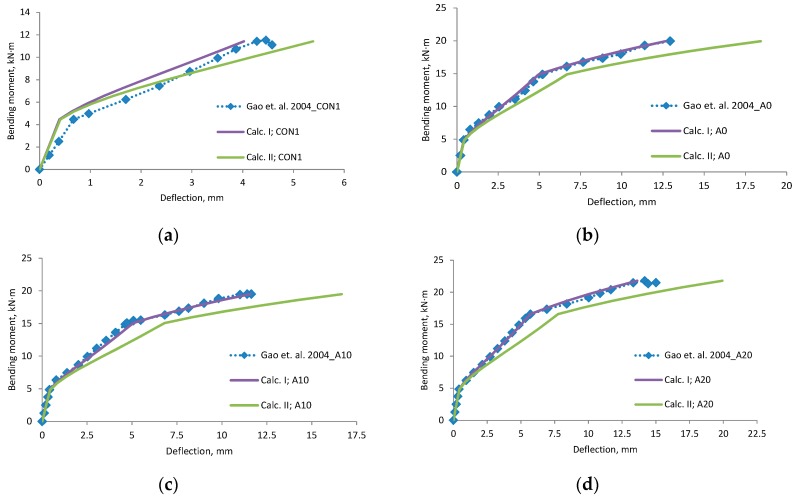

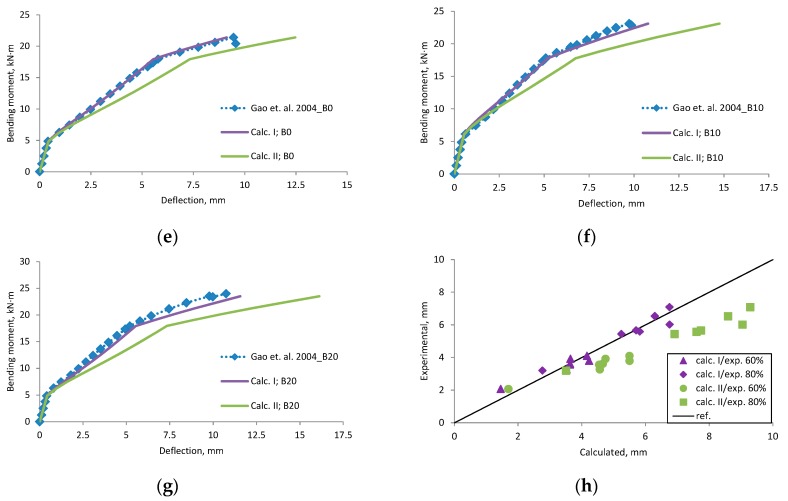
Gao et al., 2004 [29] research beams deflection compared with calculated, (**a**) beam CON1; (**b**) beam A0; (**c**) beam A10; (**d**) beam A20; (**e**) beam B0; (**f**) beam B10; (**g**) beam B20; (**h**) scatter of the results at 60% and 80% of the ultimate load.

**Figure A7:**
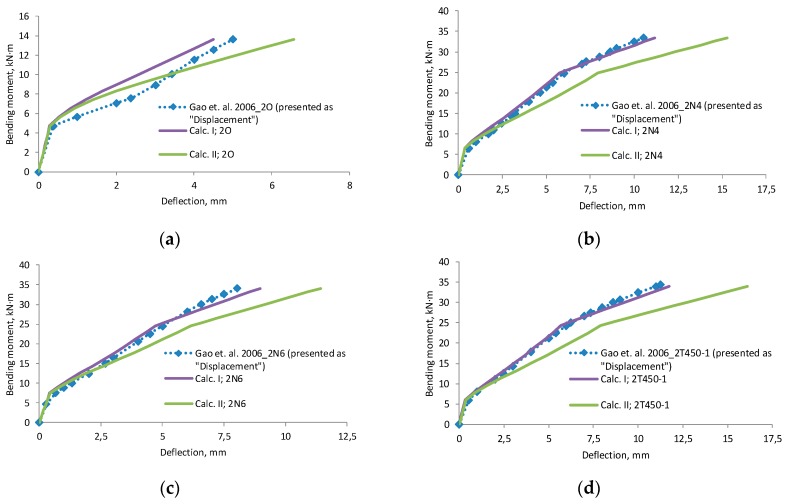

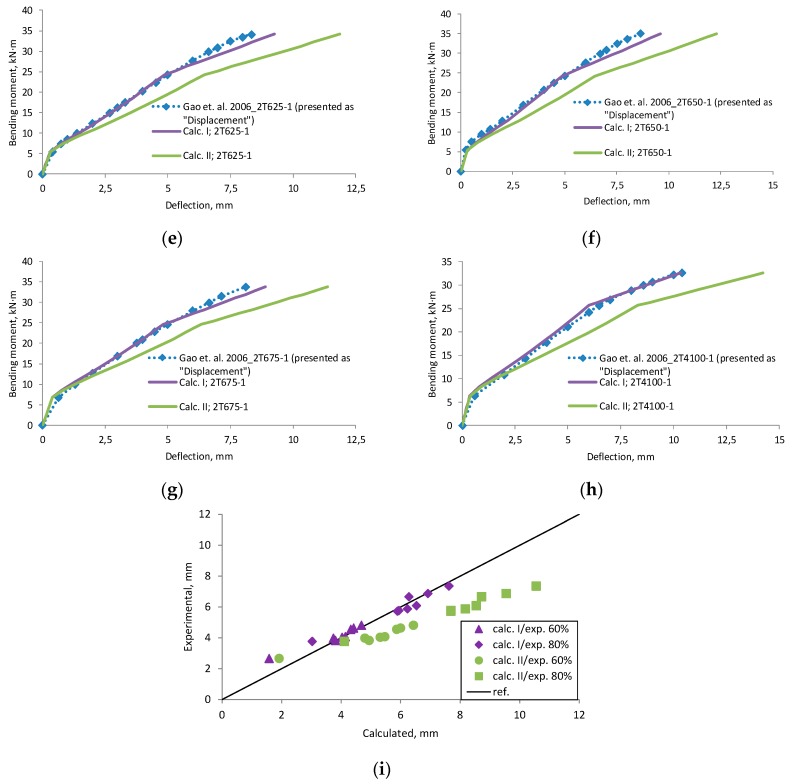
Gao et al., 2006 [30] research beams displacement compared with calculated deflection, (**a**) beam 2O; (**b**) beam 2N4; (**c**) beam 2N6; (**d**) beam 2T450-1; (**e**) beam 2T625-1; (**f**) beam 2T650-1; (**g**) beam 2T675-1; (**h**) beam 2T4100-1; (**i**) scatter of the results at 60 % and 80% of the ultimate load.

**Figure A8:**
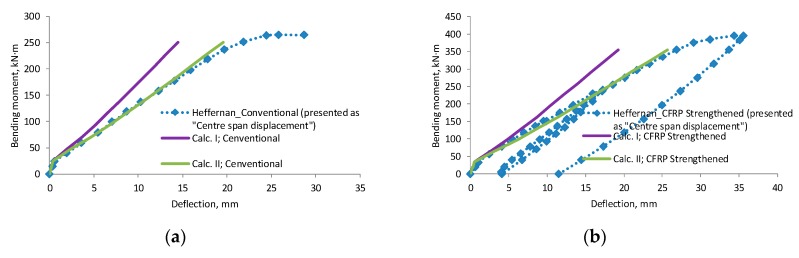

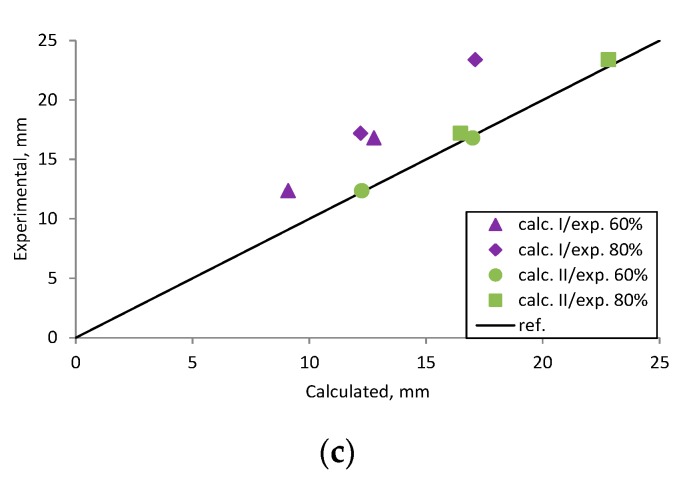
Heffernan 1997 [31] research beams displacement compared with calculated deflection, (**a**) beam Conventional; (**b**) beam CFRP Strengthened; (**c**) scatter of the results at 60% and 80% of the ultimate load.

**Figure A9:**
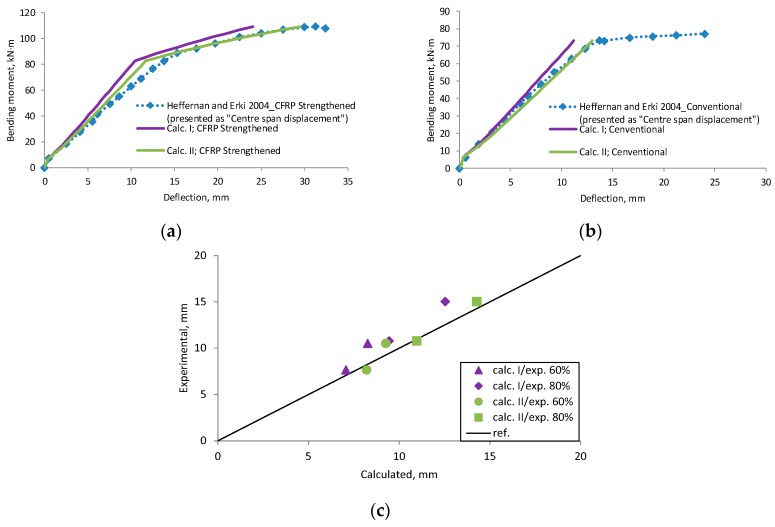
Heffernan and Erki 2004 [32] research beams displacement compared with calculated deflection, (**a**) beam CFRP Strengthened; (**b**) beam Conventional; (**c**) scatter of the results at 60% and 80% of the ultimate load.

**Figure A10:**
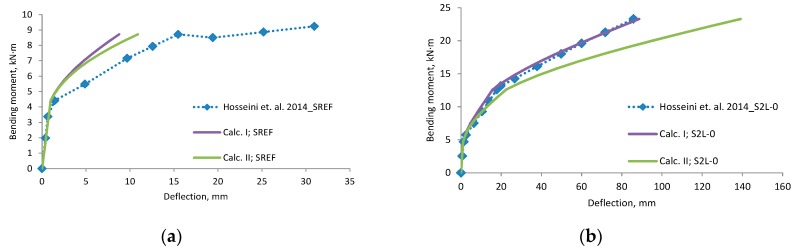

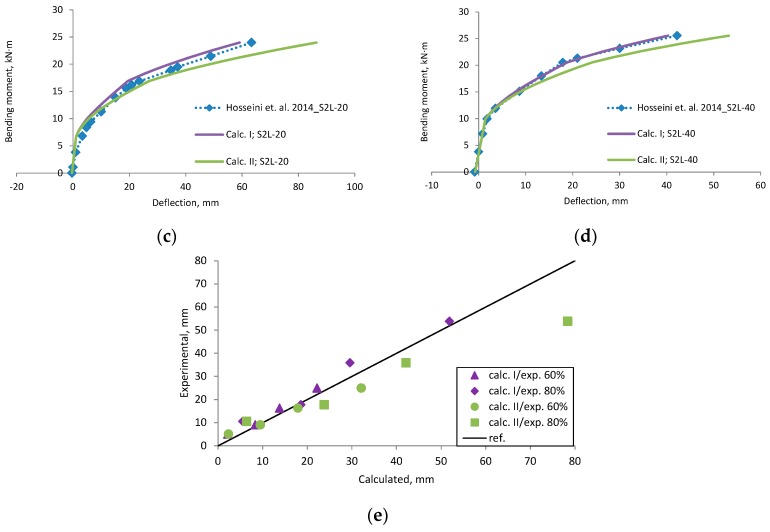
Hosseini et al., 2014 [33] research beams deflection compared with calculated, (**a**) beam SREF; (**b**) beam S2L-0; (**c**) beam S2L-20; (**d**) beam S2L-40; (**e**) scatter of the results at 60% and 80% of the ultimate load.

**Figure A11:**
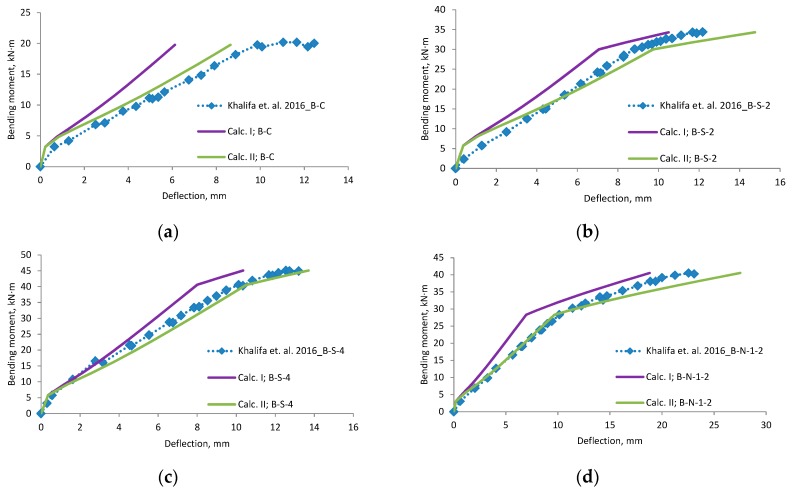

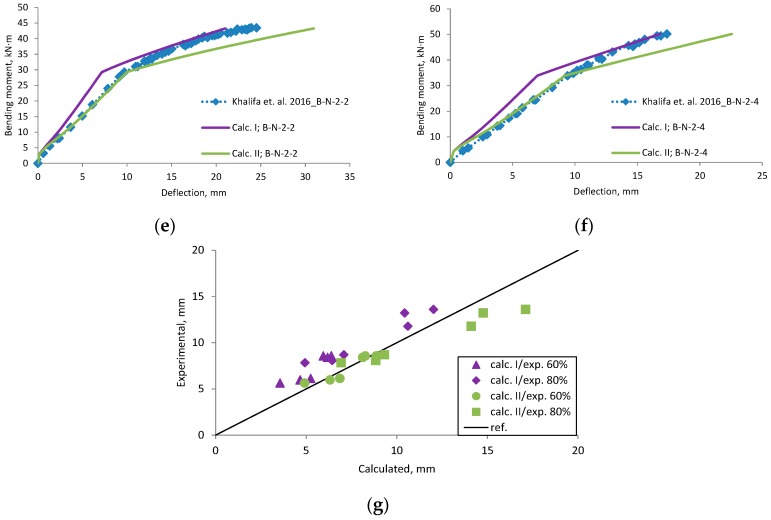
Khalifa et al., 2016 [34] research beams deflection compared with calculated, (**a**) beam B-C; (**b**) beam B-S-2; (**c**) beam B-S-4; (**d**) beam B-N-1-2; (**e**) beam B-N-2-2; (**f**) beam B-N-2-4; (**g**) scatter of the results at 60% and 80% of the ultimate load.

**Figure A12:**
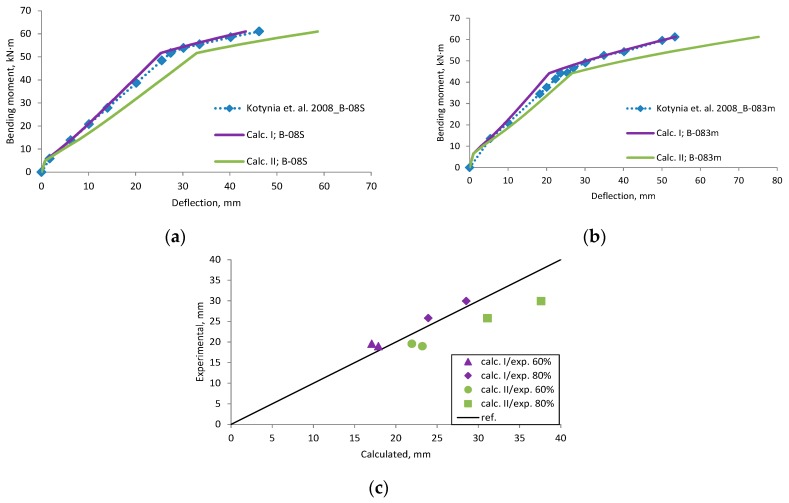
Kotynia et al., 2008 [35] research beams deflection compared with calculated, (**a**) beam B-08S; (**b**) beam B-083m; (**c**) scatter of the results at 60% and 80% of the ultimate load.

**Figure A13:**
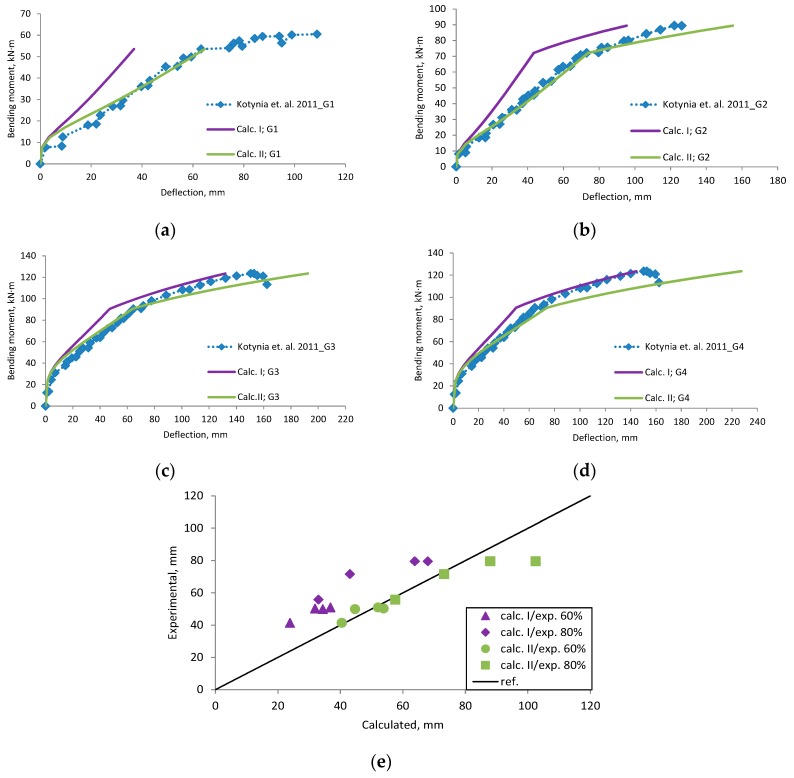
Kotynia et al., 2011 [36] research beams deflection compared with calculated, (**a**) beam G1; (**b**) beam G2; (**c**) beam G3; (**d**) beam G4; (**e**) scatter of the results at 60% and 80% of the ultimate load.

**Figure A14:**
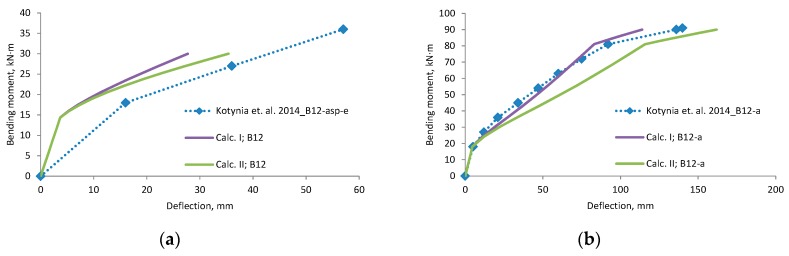

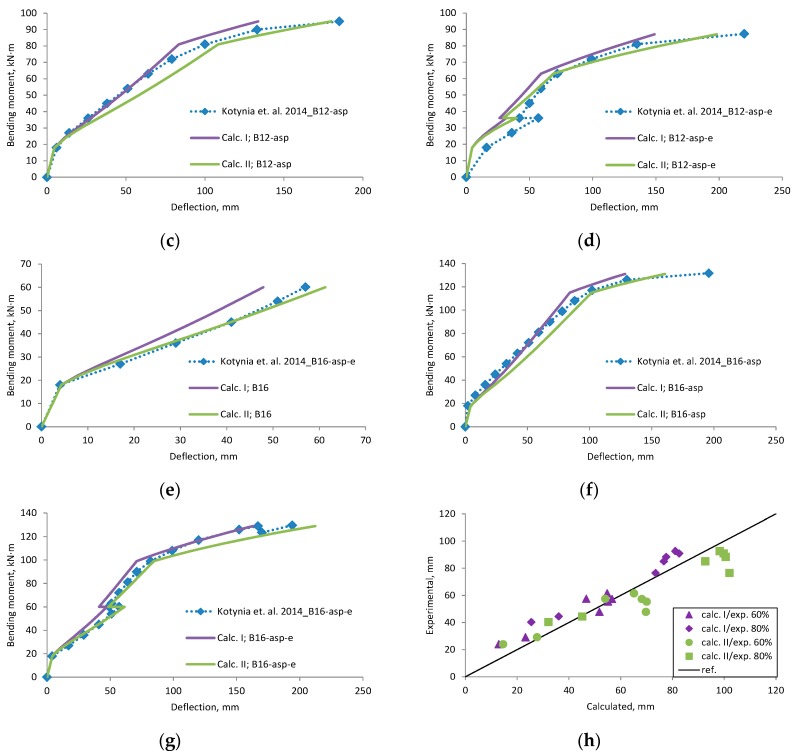
Kotynia et al., 2014 [37] research beams deflection compared with calculated, (**a**) beam B12; (**b**) beam b12-a; (**c**) beam B12-asp; (**d**) beam B12-asp-e; (**e**) beam B16; (**f**) beam B16-asp; (**g**) beam B16-asp-e; (**h**) scatter of the results at 60% and 80% of the ultimate load.

**Figure A15:**
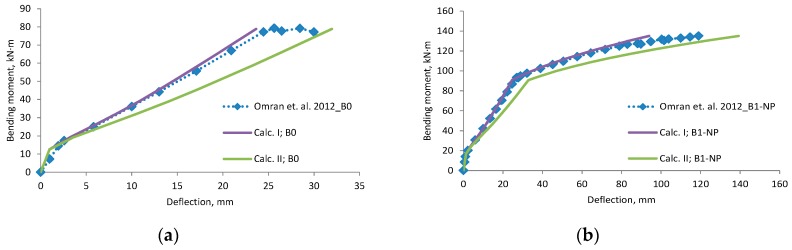

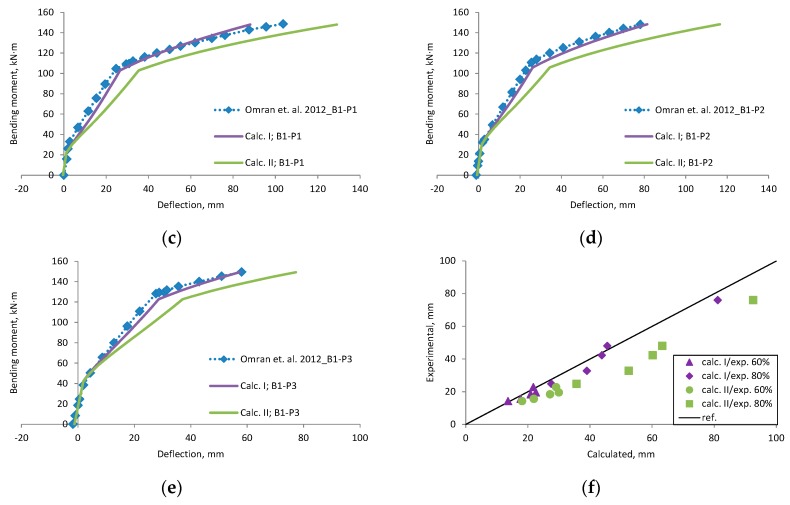
Omran et al., 2012 [38] research beams deflection compared with calculated, (**a**) beam B0; (**b**) beam B1-NP; (**c**) beam B1-P1; (**d**) beam B1-P2; (**e**) beam B1-P3; (**f**) scatter of the results at 60% and 80% of the ultimate load.

**Figure A16:**
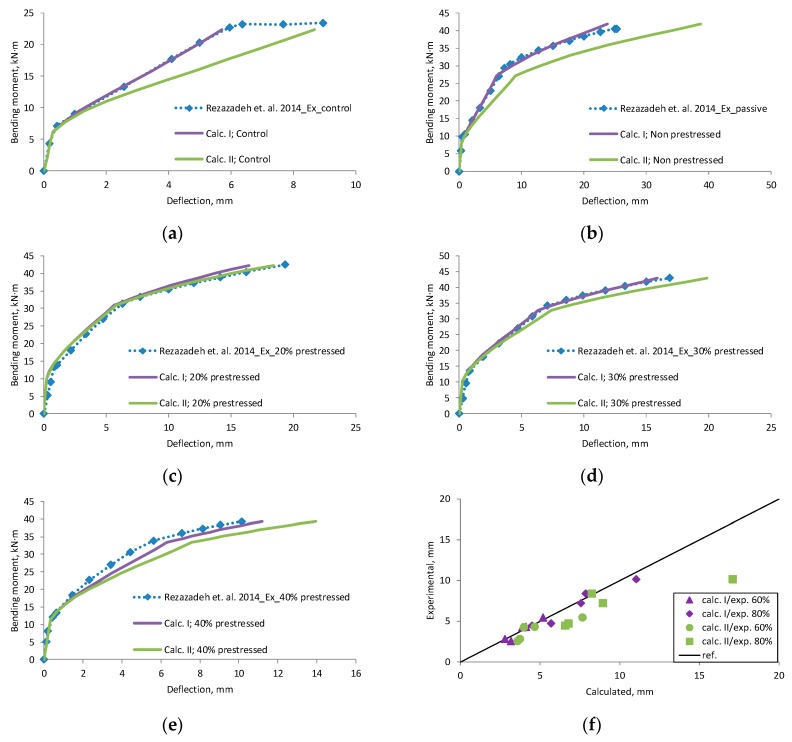
Rezazadeh et al., 2014 [39] research beams deflection compared with calculated, (**a**) beam Control; (**b**) beam Non prestressed; (**c**) beam 20% prestressed; (**d**) beam 30% prestressed; (**e**) beam 40% prestressed; (**f**) scatter of the results at 60% and 80% of the ultimate load.

**Figure A17:**
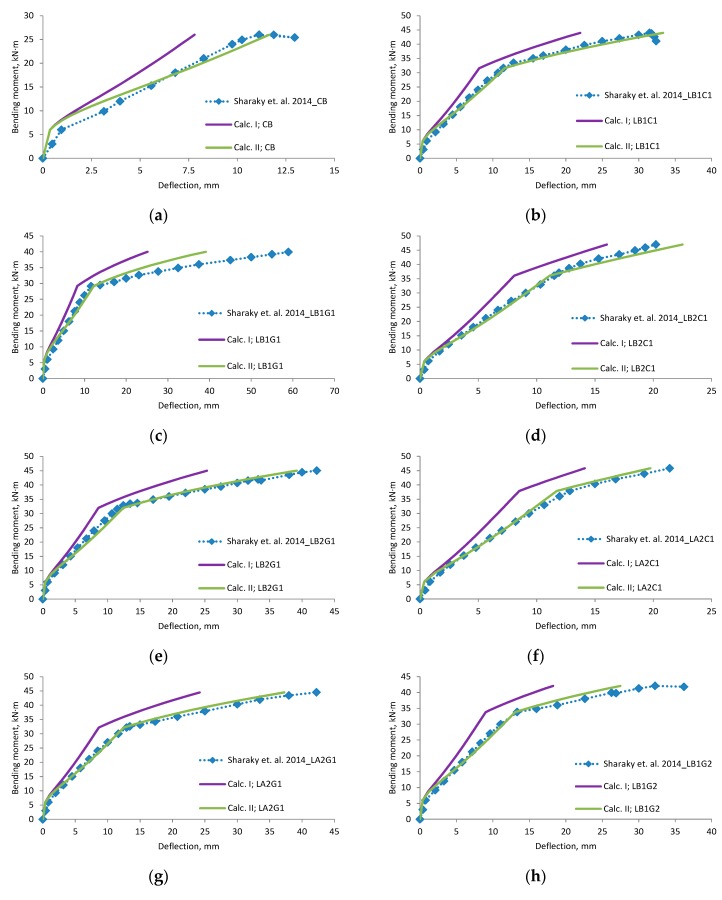

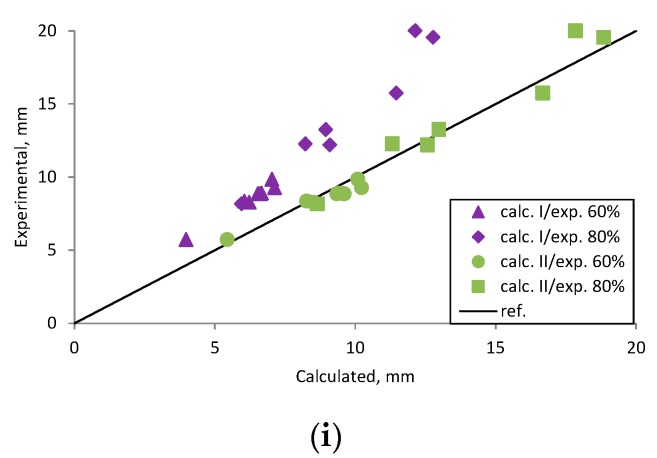
Sharaky et al., 2014 [40] research beams deflection compared with calculated, (**a**) beam CB; (**b**) beam LB1C1; (**c**) beam LB1G1; (**d**) beam LB2C1; (**e**) beam LB2G1; (**f**) beam LA2C1; (**g**) beam LA2G1; (**h**) beam LB1G2; (**i**) scatter of the results at 60% and 80% of the ultimate load.

**Figure A18:**
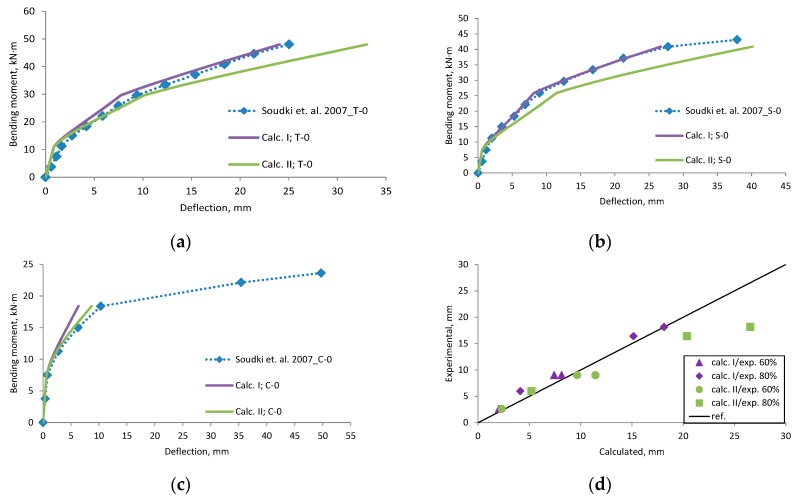
Soudki et al., 2007 [41] research beams deflection compared with calculated, (**a**) beam T-0; (**b**) beam S-0; (**c**) beam C-0; (**d**) scatter of the results at 60% and 80% of the ultimate load.

**Figure A19:**
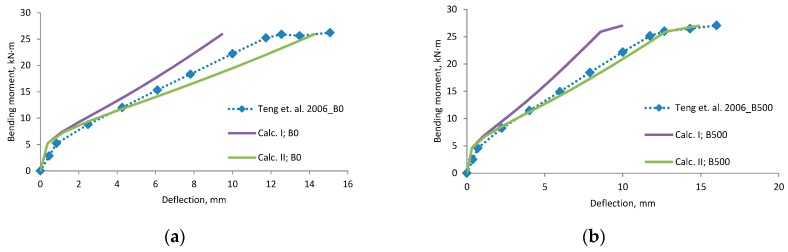

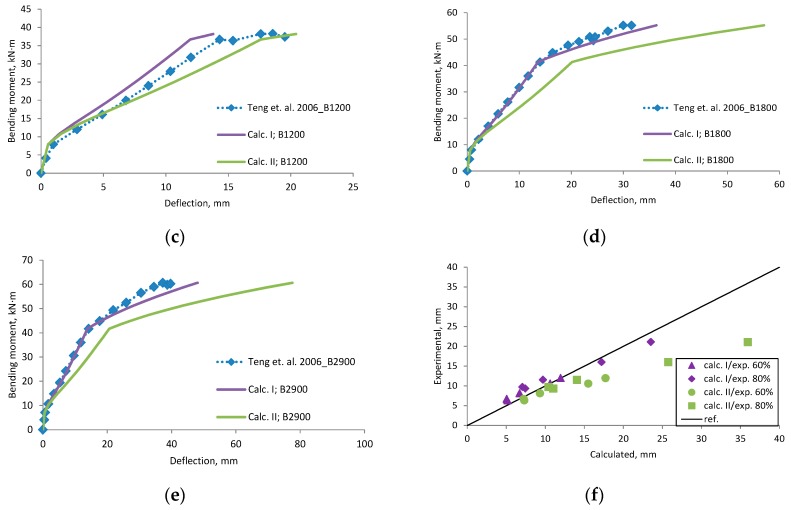
Teng et al., 2006 [42] research beams deflection compared with calculated, (**a**) beam B0; (**b**) beam B500; (**c**) beam B1200; (**d**) beam B1800; (**e**) beam B2900; (**f**) scatter of the results at 60% and 80% of the ultimate load.

**Figure A20:**
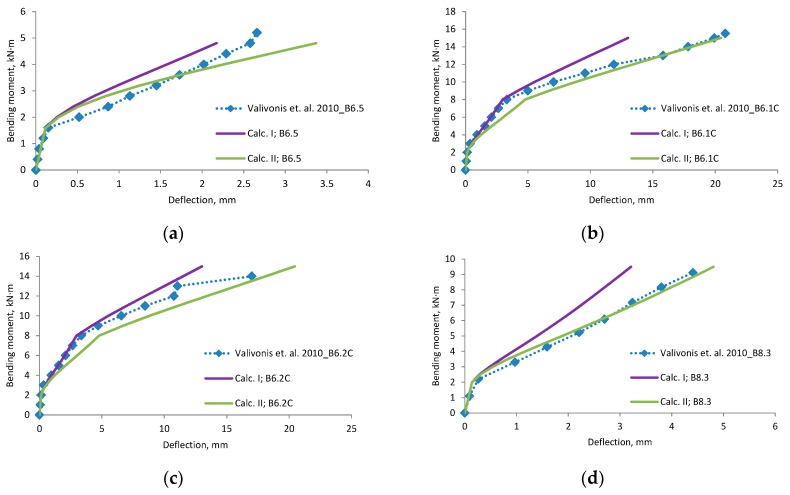

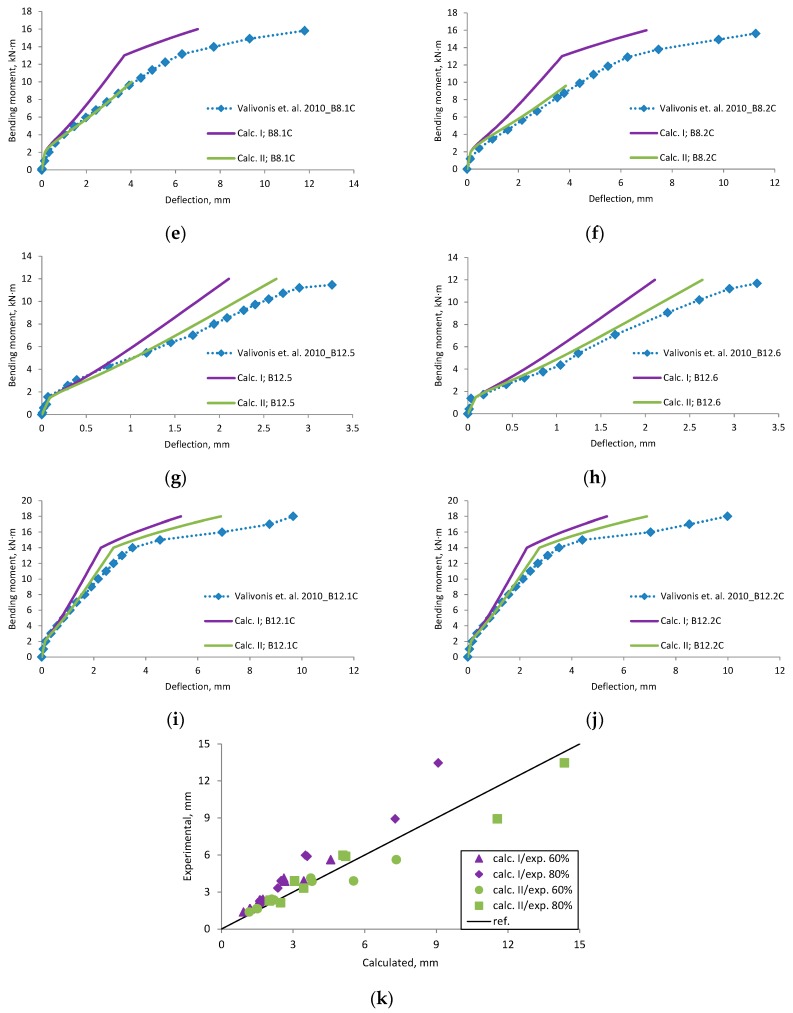
Valivonis et al., 2010 [14] research beams deflection compared with calculated, (**a**) beam B6.5; (**b**) beam B6.1C; (**c**) beam B6.2C; (**d**) beam B8.3; (**e**) beam B8.1C; (**f**) beam B8.2C; (**g**) beam B12.5; (**h**) beam B12.6; (**i**) beam B12.1C; (**j**) beam B12.2C; (**k**) scatter of the results at 60% and 80% of the ultimate load.

**Figure A21:**
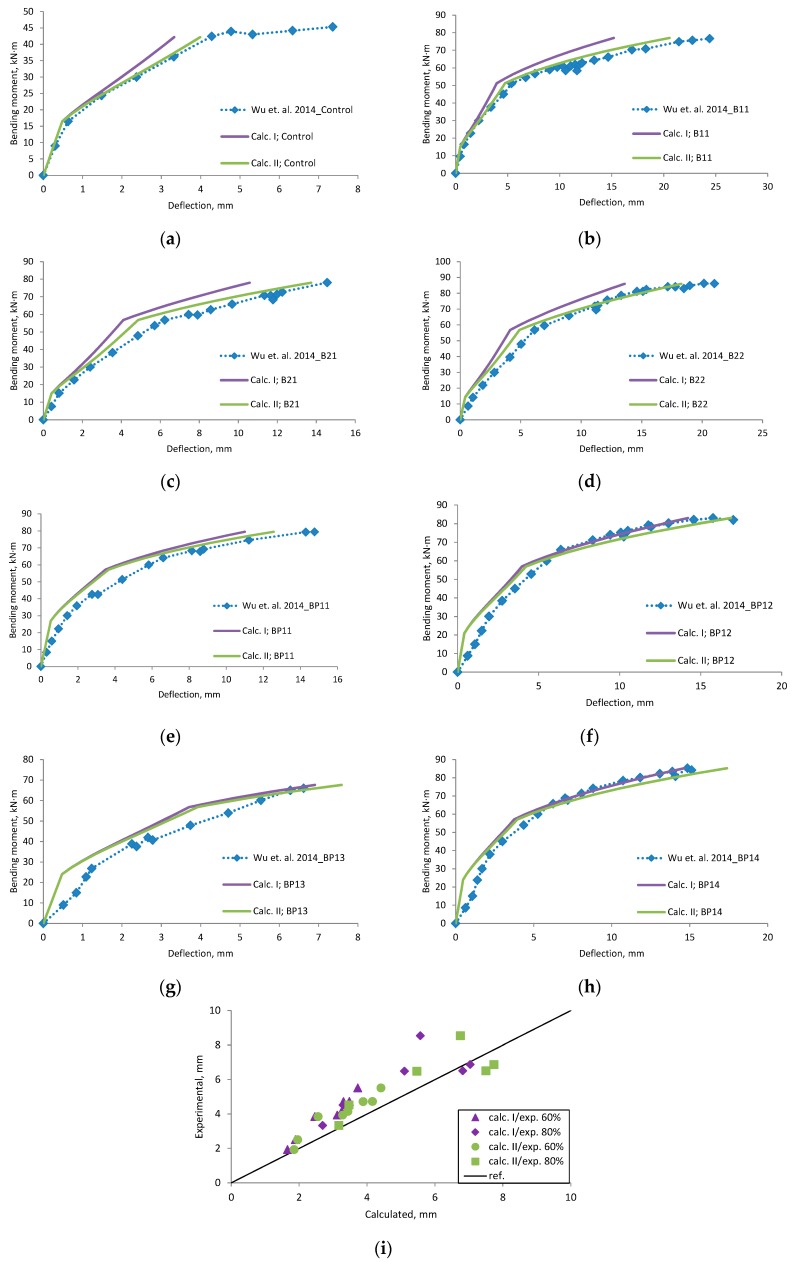
Wu et al., 2014 [43] research beams deflection compared with calculated, (**a**) beam Control; (**b**) beam B11; (**c**) beam B21; (**d**) beam B22; (**e**) beam BP11; (**f**) bam BP12; (**g**) beam BP13; (**h**) beam BP14; (**i**) scatter of the results at 60% and 80% of the ultimate load.

**Figure A22:**
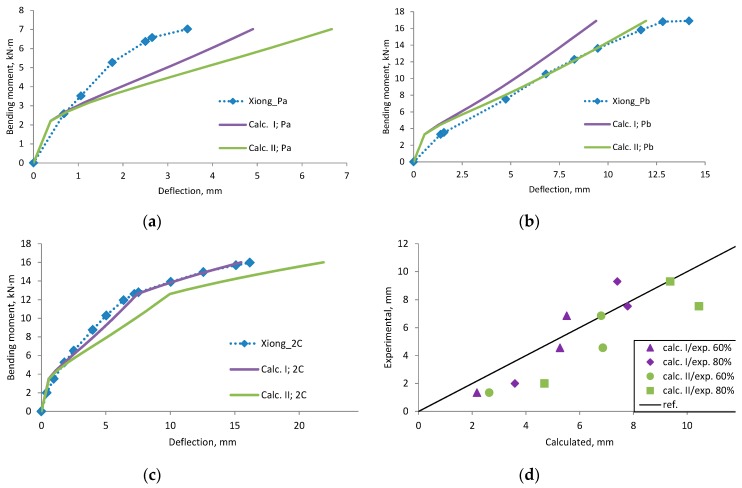
Xiong et al., 2007 [44] research beams deflection compared with calculated, (**a**) beam Pa; (**b**) beam Pb; (**c**) beam 2C; (**d**) scatter of the results at 60% and 80% of the ultimate load.

## Notation

*A*, *B*, *C*	the designation of the equation for the depth of the neutral axis;
*A_c_*	the cross section of the compressed concrete layer;
*A_f_*	the cross-section area of carbon fiber;
*A_red_*	the transformed cross section of the beam;
*A_s_*_1_ and *A_s_*_2_	the cross-section area of the tensioned and compressed reinforcement;
*E_cm_*	the modulus of elasticity of concrete;
*E_f_*	the modulus of elasticity of carbon fiber;
*E_s_*_1_, *E_s_*_2_	the modulus of elasticity of steel bars;
*I_II_*, *I_III_*	the effective moment of inertia at stages 2 and 3;
*I*_*I*.*red*_, *I*_*II*.*red*_, *I*_*III*.*red*_	the moment of the inertia of the transformed cross-section at stages 1, 2, and 3;
*M_I_* and *M*_*I*.*S*_	the cracking moment of the unstrengthened beam and strengthened beam respectively;
*M*_*II*.*S*_	the bending moment of the strengthened beam when the yielding of reinforcement is reached;
*M_R_*, *M_II_*	the maximum carrying bending moment of the unstrengthened beam;
*M*_*R*.*S*_, *M_II_*_I_	the maximum carrying bending moment of the strengthened beam;
*S_red_*	the static moment of the transformed cross-section;
*a*	distance from the support to loading;
*b*	the width of the beam;
*d*_1_ and *d*_2_	distance from the beam edge to the center of the tensioned and compressed reinforcement;
*f_c_*	the compressive strength of concrete cylinders;
*f_ct_*	the tensile strength of concrete;
*h*	the height of the beam;
*k*_1_	the coefficient evaluating the shape of stress distribution;
*l*	the span length of the beam;
*t_f_*	the thickness of the carbon fiber layer;
*x_I_*, *x_II_* and *x_III_*	the depth of the neutral axis at stages 1, 2 and 3;
*y*_*c*.*I*_, *y*_*c*.*II*_, *y*_*c*.*III*_,	the centre of the gravity of the beam cross-section at stages 1, 2 and 3;
*α_f_*, *α_s_*_1_, *α_s_*_2_	relative coefficients;
*γ*_1.*c*_, *γ*_1.*t*_	relative coefficients evaluating a change in the depth of the neutral axis at stage 2;
*γ*_2.*c*_, *γ*_2.*t*_	relative coefficients evaluating a change in the depth of the neutral axis at stage 3;
*ε_c_*	the strain of the compressed concrete;
*ε_c_*_1_	strain when the maximum strength of concrete material is reached;
*ε_f_*	the strain of the carbon fiber layer;
*ε_s_*	the strain of the tensioned reinforcement;
*ε_s_*_2_	the strain of the compressed reinforcement;
*σ_c_*	stresses in the layer of the compressed concrete;
*σ_f_*	stresses in the layer of carbon fiber;
*σ_s_*	stresses in the tensioned reinforcement;
*σ_s_*_2_	stresses in the compressed reinforcement;
*ω_I_*, *ω_II_*	the deflection of the control beam up to the end of stages I and II.
*ω*_*I*.*S*_, *ω*_*II*.*S*_, *ω*_*III*.*S*_	the deflection of the strengthened beam up to the end of stages I, II, and III.

## References

[1] Skuturna T., Valivonis J., Vainiūnas P., Marčiukaitis G., Daugevičius M. (2008). Analysis of deflections of bridge girders strengthened by carbon fibre reinforcement. Balt. J. Road Bridge Eng..

[2] Daugevičius M., Valivonis J., Marčiukaitis G. (2012). Deflection analysis of reinforced concrete beams strengthened with carbon fibre reinforced polymer under long-term load action. J. Zhejiang Univ.-Sci. A (Appl. Phys. Eng.).

[3] Skuturna T., Valivonis J. (2015). The statistical evaluation of design methods of the load-carrying capacity of flexural reinforced concrete elements strengthened with FRP. Arch. Civ. Mech. Eng..

[4] Skuturna T., Valivonis J. (2016). Experimental study on the effect of anchorage systems on RC beams strengthened using FRP. Compos. Part B.

[5] Skuturna T., Valivonis J. (2016). Evaluation of calculation methods used for estimating the ultimate moment resistance of bridge decks reinforced with FRP bars. Balt. J. Road Bridge Eng..

[6] Eslami A., Ronagh H.R., Mostofinejad D. (2016). Analytical Assessment of CFRP Retrofitted Reinforced-Concrete Buildings Subjected to Near-Fault Ground Motions. J. Perform. Constr. Facil..

[7] Al-Rousan R., Issa M. (2011). Fatigue performance of reinforced concrete beams strengthened with CFRP sheets. Constr. Build. Mater..

[8] Attari N., Amziane S., Chemrouk M. (2012). Flexural strengthening of concrete beams using CFRP, GFRP and hybrid FRP sheets. Constr. Build. Mater..

[9] Li X., Gu X., Song X., Ouyang Y., Feng Z. (2013). Contribution of U-shaped strips to the flexural capacity of low-strength reinforced concrete beams strengthened with carbon fibre composite sheets. Compos. Part B.

[10] Charalambidi B.G., Rousakis T.C., Karabinis A.I. (2016). Analysis of the fatigue behavior of reinforced concrete beams strengthened in flexure with fiber reinforced polymer laminates. Compos. Part B.

[11] Triantafyllou G.G., Rousakis T.C., Karabinis A.I. (2017). Corroded RC beams patch repaired and strengthened in flexure with fiber-reinforced polymer laminates. Compos. Part B.

[12] Charalambidi B.G., Rousakis T.C., Karabinis A.I. (2016). Fatigue Behavior of Large-Scale Reinforced Concrete Beams Strengthened in Flexure with Fiber-Reinforced Polymer Laminates. J. Compos. Constr..

[13] Zhang S.S., Yu T., Chen G.M. (2017). Reinforced concrete beams strengthened in flexure with near-surface mounted (NSM) CFRP strips: Current status and research needs. Compos. Part B.

[14] Valivonis J., Skuturna T., Daugevičius M. The load-carrying capacity of reinforced concrete beams strengthened with carbon fibre composite in the tension zone subjected to temporary or sustained load. Proceedings of the 10th International Conference on Modern Building Materials. Structures and Techniques.

[15] Hawileh R.A. (2012). Nonlinear finite element modelling of RC beams strengthened with NSM FRP rods. Constr. Build. Mater..

[16] ACI Committee 318 (2002). Building Code Requirement for Structural Concrete.

[17] (2004). Eurocode 2: Design of Concrete Structures—Part 1: General Rules and Rules for Buildings.

[18] Paththini M.M., Burgoyne A., Burgoyne C. (2009). Moment-Curvature and Strain energy of Beams with External Fiber-Reinforced Polymer Reinforcement. ACI Struct. J..

[19] Yinghao L., Yong Y. (2013). Arrangement of hybrid rebars on flexural behavior of HSC beams. Compos. Part B.

[20] Guan G.X., Burgoyne C.J. (2014). Comparison of Moment-Curvature Models for Fiber-Reinforced Polymer Plate-End Debonding Studies Using Global Energy Balance Approach. ACI Struct. J..

[21] Rezazadeh M., Barros J., Costa L. (2015). Analytical approach for flexural analysis of RC beams strengthened with prestressed CFRP. Compos. Part B.

[22] Smith S.T., Kim S.J. (2010). Deflection Calculation of Frp-Strengthened Reinforced Concrete Flexural Members. Aust. J. Struct. Eng..

[23] Smith S.T., Rasheed H.A., Kim S.J. Moment-Curvature Based Modeling of FRP-Strengthened RC Members Anchored with FRP Anchors. Proceedings of the APFIS.

[24] Barros J.A.O., Fortes A.S. (2005). Flexural strengthening of concrete beams with CFRP laminates bonded into slits. Cem. Concr. Compos..

[25] Bilotta A., Ceroni F., Nigro E., Pecce M. (2015). Efficiency of CFRP NSM strips and EBR plates for flexural strengthening of RC beams and loading pattern influence. Compos. Struct..

[26] David E., Ragneau E., Buyle-Bodin E. (2003). Experimental analysis of flexural behaviour of externally bonded CFRP reinforced concrete structures. Mater. Struct..

[27] El-Gamal S.E., Al-Nuaimi A. (2016). Efficiency of near surface mounted technique using fiber reinforced polymers for the flexural strengthening of RC beams. Constr. Build. Mater..

[28] Ferrier E., Avril A., Hamelin P., Vautrin A. (2003). Mechanical behavior of RC beams reinforced by externally bonded CFRP sheets. Mater. Struct..

[29] Gao B., Kim J.K., Leung C.K.Y. (2004). Experimental study on RC beams with FRP strips bonded with rubber modified resins. Compos. Sci. Technol..

[30] Gao B., Kim J.K., Leung C.K.Y. (2006). Strengthening efficiency of taper ended FRP strips bonded to RC beams. Compos. Sci. Technol..

[31] Heffernan P.J. (1997). Fatigue behaviour of reinforced concrete beams strengthened with CFRP laminates. Ph.D. Thesis.

[32] Heffernan P.J., Erki M.A. (2004). Fatigue Behavior of Reinforced Concrete Beams Strengthened with Carbon Fiber Reinforced Plastic Laminates. J. Compos. Constr..

[33] Hosseini M.R.M., Dias S.J.E., Barros J.A.O. (2014). Effectiveness of prestressed NSM CFRP laminates for the flexural strengthening of RC slabs. Compos. Struct..

[34] Khalifa A.M. (2016). Flexural performance of RC beams Strengthened with near surface mounted CFRP strips. Alex. Eng. J..

[35] Kotynia R., Baky H.A., Neale K.W., Ebead U.A. (2008). Flexural Strengthening of RC Beams with Externally Bonded CFRP Systems: Test Results and 3D Nonlinear FE Analysis. J. Compos. Constr..

[36] Kotynia R., Walenziak R., Stoecklin I., Meier U. (2011). RC Slabs Strengthened with Prestressed and Gradually Anchored CFRP Strips under Monotonic and Cyclic Loading. J. Compos. Constr..

[37] Kotynia R., Lasek K., Staskiewicz M. (2014). Flexural Behavior of Preloaded RC Slabs Strengthened with Prestressed CFRP Laminates. J. Compos. Constr..

[38] Omran H.Y., El-Hacha R. (2012). Nonlinear 3D finite element modeling of RC beams strengthened with prestressed NSM-CFRP strips. Constr. Build. Mater..

[39] Rezazadeh M., Costa I., Barros J. (2014). Influence of prestress level on NSM CFRP laminates for the flexural strengthening of RC beams. Compos. Struct..

[40] Sharaky I.A., Torres L., Comas J., Barris C. (2014). Flexural response of reinforced concrete (RC) beams strengthened with near surface mounted (NSM) fiber reinforced polymer (FRP) bars. Compos. Struct..

[41] Soudki K., El-Salakawy E., Craig B. (2007). Behavior of CFRP Strenghtened Rainforced Concrete Beams in Corosive Environment. J. Compos. Constr..

[42] Teng J.G., De Lorenzis L., Wang B., Li R., Wong T.N., Lam L. (2006). Debonding Failures of RC Beams Strengthened with Near Surface Mounted CFRP Strips. J. Compos. Constr..

[43] Wu G., Dong Z.Q., Wu Z.S., Zhang L.W. (2014). Performance and Parametric Analysis of Flexural Strengthening for RC Beams with NSM-CFRP Bars. J. Compos. Constr..

[44] Xiong G.J., Jiang X., Liu J.W., Chen L. (2007). A way for preventing tension delamination of concrete cover in midspan of FRP strengthened beams. Constr. Build. Mater..

[45] Branson D.E. (1977). Deformation of Concrete Structures.

